# Unveiling Dynamic System Strategies for Multisensory Processing: From Neuronal Fixed-Criterion Integration to Population Bayesian Inference

**DOI:** 10.34133/2022/9787040

**Published:** 2022-08-19

**Authors:** Jiawei Zhang, Yong Gu, Aihua Chen, Yuguo Yu

**Affiliations:** ^1^State Key Laboratory of Medical Neurobiology and MOE Frontiers Center for Brain Science, Shanghai Artificial Intelligence Laboratory, Research Institute of Intelligent and Complex Systems and Institute of Science and Technology for Brain-Inspired Intelligence, Human Phenome Institute, Shanghai 200433, China; ^2^Key Laboratory of Primate Neurobiology, Institute of Neuroscience, CAS Center for Excellence in Brain Science and Intelligence Technology, Chinese Academy of Sciences, Shanghai, China; ^3^Key Laboratory of Brain Functional Genomics (Ministry of Education), East China Normal University, 3663 Zhongshan Road N., Shanghai 200062, China

## Abstract

Multisensory processing is of vital importance for survival in the external world. Brain circuits can both integrate and separate visual and vestibular senses to infer self-motion and the motion of other objects. However, it is largely debated how multisensory brain regions process such multisensory information and whether they follow the Bayesian strategy in this process. Here, we combined macaque physiological recordings in the dorsal medial superior temporal area (MST-d) with modeling of synaptically coupled multilayer continuous attractor neural networks (CANNs) to study the underlying neuronal circuit mechanisms. In contrast to previous theoretical studies that focused on unisensory direction preference, our analysis showed that synaptic coupling induced cooperation and competition in the multisensory circuit and caused single MST-d neurons to switch between sensory integration or separation modes based on the fixed-criterion causal strategy, which is determined by the synaptic coupling strength. Furthermore, the prior of sensory reliability was represented by pooling diversified criteria at the MST-d population level, and the Bayesian strategy was achieved in downstream neurons whose causal inference flexibly changed with the prior. The CANN model also showed that synaptic input balance is the dynamic origin of neuronal direction preference formation and further explained the misalignment between direction preference and inference observed in previous studies. This work provides a computational framework for a new brain-inspired algorithm underlying multisensory computation.

## 1. Introduction

The primate brain frequently combines multisensory information from different sensory modalities, such as information of visual, vestibular, auditory, and haptic origin, to improve the perception of the external world. Both visual and vestibular information is valuable for the multisensory cortex to infer self-motion and object motion direction accurately in real time. Previous experimental studies [1, 2] showed that the macaque dorsal medial superior temporal region (MST-d) contains neurons responsible for multisensory encoding, e.g., vestibular and visual motion cues. Experimental studies observed that some MST-d neurons respond preferably to vestibular and visual motion in the same direction (called “congruent” neurons), while others prefer opposing directions (called “opposite” neurons) [1–4]. Recent theoretical studies suggested that congruent neurons mainly implement cue integration, while opposite neurons mainly perform segregation. The responses of the congruent and opposite neurons are critical for the animal to make inference about whether information from the visual and vestibular senses are attributed to a common source or two separated ones. However, the mechanism for the origin of congruent or opposite neurons in MST circuit is rarely studied. Moreover, the mechanisms through which neurons implement multisensory integration and separation are also debated [5–13].

Since sensory signals vary across modalities and conditions, Ernst and Banks proposed a general principle that the brain determines the degree to which a sense dominates the flow of information based on its reliability, which is defined as the variance of the sensory estimate [14]. Further works demonstrated that human and monkey subjects adjusted the weight of each sensor based on cue reliability (stimulus motion coherence) and made the decisions about motion directions in a near-optimal way [15–18]. Evidence from experiments suggested that multisensory cortical neurons, e.g., MST-d (dorsal medial superior temporal), could represent the weighing of cue reliability by the neural response [3], comprising a link between single-neuron activity and behavior. Combining the theories and behavioral data, many works assumed that the neural system performs causal inference in Bayesian approach [19–21], which redefines the inference problem as an assessment of the posterior probability of integration based on the measurement distribution of each cue. The Bayesian causal inference (BCI) model managed to explain the experimental findings that spatially concurrent visual and vestibular inputs improved direction discrimination performance [3], which is mainly attributed to congruent neurons [4], while spatially confined inputs are canceled out by opposing neurons [22, 23]. Furthermore, by fitting the distribution width of sensory measurements, Bayesian computation captures the varying uncertainty that originates from both stimuli contrast and physiological noise [24]. Nonetheless, the BCI model does not explain the biophysical computation principle in neural systems due to the limit of its mathematical form. It remains to be solved how the Bayesian approach is achieved physically by neural systems, especially how the prior and posterior probability is represented by neuronal firing.

In this study, we seek to combine both physiological recordings and computational models and explore two key scientific questions: (1) How are visual and vestibular signals integrated and separated by neuronal circuits? (2) How do multisensory computing algorithms emerge from hierarchical cortical circuits for behavioral-level inference decisions? In contrast to previous works that attribute the inference to the interaction between the multisensory areas [13], this study first focuses on the multisensory computation of each MST-d neuron, which plays a more fundamental role. By investigating the physiological data from monkey MST-d neurons, we found that neuronal direction preference in the MST-d is correlated with relative synaptic input strength between the visual and vestibular inputs, which may entail multisensory computation. We further built a multipartite cortical circuit model that is composed of three continuous attractor neural networks (CANNs) [25]. The circuit consists of three ring attractor networks, mimicking input transmission from the unisensory visual and vestibular regions to the MST-d along the cortical hierarchy. We demonstrate by this model that MST-d neurons naturally compose the coding bases for integration or separation by various synaptic coupling strengths between the inputs. The change of direction preference as observed in the data is an explicit form of the nonlinear coupling dynamic.

Next, we went a further step and applied this computational principle to hypothetical decisions about whether the inputs should be integrated. We revealed that individual MST-d neurons implement a fixed-criterion strategy, which makes decisions with deterministic boundaries. However, by pooling MST-d neurons with different synaptic coupling strengths, the MST-d population implements Bayesian inference that leads to distinct decisions based on cue reliability, constituting a bioplausible process for multisensory inference.

## 2. Results

### 2.1. Analysis of Physiological Data

We began with physiological recordings. We first characterized MST-d neurons with their tuning response functions to the input senses. The MST region is not only a crucial multisensory region (responding to both visual and vestibular inputs [1, 26]) but also correlates with perception at the behavioral level [4, 27, 28]. Specifically, the dorsal part of the MST has a large receptive field that can respond to translational motion signals [29], which is well suited for detecting self-motion and object motion features in the horizontal plane.

**Figure 1:**
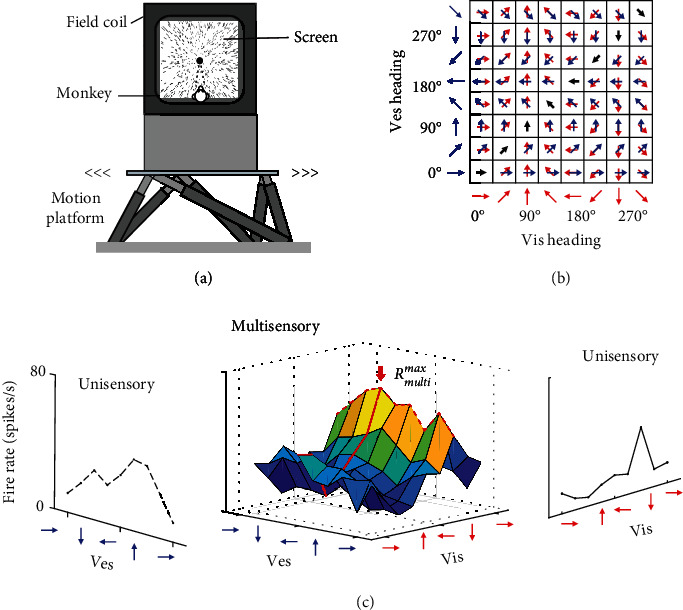
Experimental protocol and multisensory preference schematics. (a) Schematic of the physiological experiment. The screen was placed on a platform with 6 degrees of motion. Visual motion stimuli were simulated by the movement of a dot cloud, and vestibular motion stimuli were simulated by the movement of the platform. (b) Systematic table of multisensory stimuli. Each stimulus took 8 discretized movement directions spaced by 45°; thus, 64 neural responses were recorded for each neuron under the multisensory condition. (c) Neuronal responses in unisensory (side panels) and multisensory (middle panel) conditions. Multisensory preference was identified from the maximum response (*R*_multi_^max^) in the grid of 64 responses that specifies a joint visual and vestibular preferred direction. Multisensory tuning curves were identified by fixing the direction of one modality at the preferred value while varying the other (multisensory visual curve: solid red line, multisensory vestibular curve: dashed red line). Both multisensory tuning curves intersect at the maximal response *R*_multi_^max^.

In our experiments, monkeys were seated on a motion platform attached to a screen (Figure 1(a)). Without reporting, the subjects perceived visual motion through the optical flow presented on the screen or/and vestibular motion through the translation of the platform in the horizontal plane, comprising unisensory or multisensory conditions. Both visual and vestibular motion cues are designed to represent the same velocity and acceleration from one of 8 directions with 45° intervals. The multisensory condition contains 64 combinations of visual and vestibular input directions (Figure 1(b)), while the unisensory conditions contain 8 directions of each input. MST-d neuronal activities were recorded by a single-unit technique during the delivery of visual and/or vestibular stimuli. Figure 1(c) shows the response function of an example MST-d neuron in the unisensory condition (side panels) or multisensory condition (middle panel). Note that the unisensory condition means that either visual or vestibular cues are presented without the other cue (visual-only or vestibular-only), and the unisensory response is a function of each cue direction (*θ*).

(1) $$\begin{array}{c} R_{\text{vis}}^{\max}=\max\left[f_{\text{vis}}\left(\theta_{\text{vis}}\right)\right],\\ R_{\text{ves}}^{\max}=\max\left[f_{\text{ves}}\left(\theta_{\text{ves}}\right)\right], \end{array}$$

where *θ*_vis_ and *θ*_ves_ represent the visual and vestibular cue directions, *f*_vis_ and *f*_ves_ are the neuronal spatial tuning response functions, and *R*_vis_^max^ and *R*_ves_^max^ are neuronal maximal responses to either visual or vestibular cues, respectively, across all directions. We categorized each MST-d neuron by the balanced or imbalanced response level based on the ratio (*r*)

(2) $$\begin{array}{c} r=\frac{\max\left(R_{\text{vis}}^{\max},R_{\text{ves}}^{\max}\right)}{\min\left(R_{\text{vis}}^{\max},R_{ves}^{\max}\right)}. \end{array}$$

**Figure 2:**
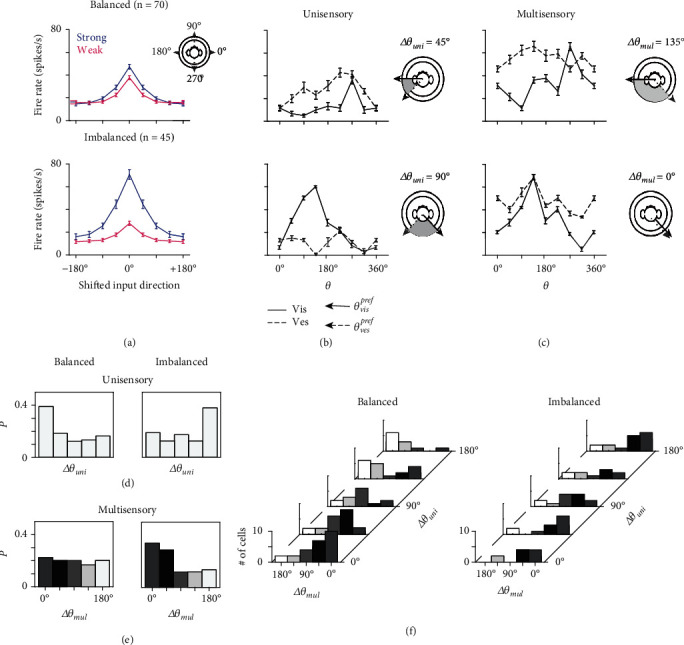
Data analysis of balanced and imbalanced neuronal preference. (a) Averaged tuning curves of balanced neurons (top panels, *n* = 70) and imbalanced neurons (bottom panels, *n* = 45) defined by the response ratio (*r*). Preferred directions are shifted to align at 0°. The (b) unisensory and (c) multisensory tuning curves of a typical balanced (top panel) or imbalanced (bottom panel) neuron. The two curves denote that fixing visual direction are preferred direction and changing vestibular direction (multisensory vestibular curve) and switching the roles as multisensory visual curve. The two curves intersect at *R*_multi_^max^ as the red lines in Figure 1(c) middle panel. Insets show the preferred visual and vestibular motion directions of the neuron as vectors, and the shaded region indicates the preference disparity. (d) Distribution of the unisensory disparity *p*(Δ*θ*_uni_) of neurons in the balanced (left) and imbalanced (right) groups. (e) Distribution of multisensory disparity *p*(Δ*θ*_mul_). (f) Joint distribution of Δ*θ*_uni_ and Δ*θ*_mul_ for balanced and imbalanced neurons. Color gradients indicate Δ*θ*_mul_.

By definition, *r* ≥ 1. To make it simple, we defined a neuron as balanced neuron when 1 ≤ *r* ≤ 1.7 and as imbalanced when *r* ≥ 1.7 (the criteria *r* = 1.7 are explained later). In the data, 70 of 115 MST-d neurons were identified as balanced neurons, characterized by relatively balanced synaptic inputs (Figure 2(a) top panel), and the average max(*R*_vis_^max^, *R*_ves_^max^) was 1.28 times the value of min(*R*_vis_^max^, *R*_ves_^max^) for neurons in this group. On the other hand, 45 of 115 neurons were identified as imbalanced neurons, characterized by the dominance of one input over the other (Figure 2(a) bottom panel). The value of max(*R*_vis_^max^, *R*_ves_^max^) in this group was 2.35 times that of min(*R*_vis_^max^, *R*_ves_^max^) on average. Since the cues were applied with the same reliability (velocity and acceleration), the ratio *r* indeed measures the degree of contribution from one sense over the other in each neuron. It was proved that response amplitudes encode the input reliability that is linked to the signal-to-noise ratio [3]; thus, *r* is a neuronal precoded bias property of cue reliability, which is independent of real-time stimuli.

Following the classification, the two encoding bases contained distinct tuning weights in unisensory condition. To specify the encoding properties in multisensory condition where both cues are presented, we examined the neural response *R*_mul_ that is a function of both cue directions *θ*_vis_ and *θ*_ves_ (Figure 1(c) middle panel)

(3) $$\begin{array}{c} R_{\text{mul}}=f_{\text{mul}}\left(\theta_{\text{vis}},\theta_{\text{ves}}\right), \end{array}$$

where *f*_mul_ is the multisensory tuning curve function. *R*_mul_^max^ = max(*R*_mul_) on the response contour is appointed to the maximal neuronal response to a specific pair of visual and vestibular directions (denoted as *θ*_vis,mul_^pref^ and *θ*_ves,mul_^pref^). We defined the spatial disparity between the two directions as a multisensory-preferred disparity.

(4) $$\begin{array}{c} \left[\theta_{\text{vis},\text{mul}}^{\text{pref}},\theta_{\text{ves},\text{mul}}^{\text{pref}}\right]=\text{argmax}\left(R_{\text{mul}}\right),\\ ∆\theta_{\text{mul}}=\min\left(\left|\theta_{\text{vis},\text{mul}}^{\text{pref}}-\theta_{\text{ves},\text{mul}}^{\text{pref}}\right|,360^{{}^{\circ}}-\left|\theta_{\text{vis},\text{mul}}^{\text{pref}}-\theta_{\text{ves},\text{mul}}^{\text{pref}}\right|\right). \end{array}$$

Similarly, the unisensory-preferred disparity was defined by the spatial disparity between the preferred directions of visual and vestibular cues (*θ*_vis,uni_^pref^ = argmax(*R*_vis_), *θ*_ves,uni_^pref^ = argmax(*R*_ves_)).

(5) $$\begin{array}{c} ∆\theta_{\text{uni}}=\min\left(\left|\theta_{\text{vis},\text{uni}}^{\text{pref}}-\theta_{\text{ves},\text{uni}}^{\text{pref}}\right|,360^{{}^{\circ}}-\left|\theta_{\text{vis},\text{uni}}^{\text{pref}}-\theta_{\text{ves},\text{uni}}^{\text{pref}}\right|\right). \end{array}$$

Both ∆*θ*_uni_ and ∆*θ*_mul_ range from 0° to 180°. In contrast to ∆*θ*_mul_, ∆*θ*_uni_ denotes the neuronal preference in the absence of multisensory interaction. As a result, ∆*θ*_uni_ is interpreted as the synaptic input disparity to each neuron, which represents the topological distance between the inherent preferences of two independent cues after synaptic learning.

Crucially, we observed that the multisensory preferences for visual and vestibular directions are usually different for each MST-d neuron, *i.e*., *θ*_vis,uni_^pref^ ≠ *θ*_vis,mul_^pref^ or *θ*_ves,uni_^pref^ ≠ *θ*_ves,mul_^pref^ (62/70 or 88.57% for balanced neurons; 40/45 or 88.89% for imbalanced neurons). As a result, ∆*θ*_mul_ is usually different from ∆*θ*_uni_ in each neuron, which revealed the effect of multisensory operation on the encoding dynamics. Intuitively, ∆*θ*_mul_ is interpreted as the disparity between the preferred directions of two sensory cues. The top panels of Figures 2(b) and 2(c) present a typical balanced neuron that the disparity between the preferred directions increased when switching from the unisensory to multisensory condition. The bottom panels of Figures 2(b) and 2(c) present an example imbalanced neuron in which the preferred disparity exhibited a decrement. The disparity preference change in multisensory condition resulted from the shifted peak of tuning curves (multisensory tuning curves are derived from red lines in Figure 1(c)). The neural mechanism will be explained by a computational neural network in the next section.

To specify the disparity preference at the population level, we investigated the probability distribution of unisensory (Figure 2(d)) and multisensory preferences (Figure 2(e)) in both balanced (left panels) and imbalanced neurons (right panel). The unisensory condition was characterized by polarized distributions. Balanced neurons generally preferred small ∆*θ*_uni_ values, while imbalanced neurons generally preferred large ∆*θ*_uni_ values. The summed population of MST-d thus had a bipolar distribution that parallels previous report [1]. Under multisensory conditions, imbalanced neurons presented a major transition to preferring a small disparity between both cues. On the other hand, balanced neurons featured a uniform-distributed preference. We further specified the relationship between ∆*θ*_uni_ and ∆*θ*_mul_, as shown in Figure 2(f). It is clear that balanced neurons had a stronger correlation between the two disparities. A small ∆*θ*_uni_ generally led to a small ∆*θ*_mul_ and vice versa. In contrast, imbalanced neurons had a weaker correlation, and ∆*θ*_mul_ was generally biased to 0° regardless of ∆*θ*_uni_.

Based on the results above, we hypothesize that imbalanced neurons may serve as a sensory integration encoding basis because (1) it is commonly acknowledged that animals are inclined to integrate stimuli with small spatial disparity [20], in which imbalanced neurons are more likely to respond strongly given the dominance of the small ∆*θ*_mul_ value. (2) If one cue is unreliable, the subject tends to integrate the cues by giving larger weights to the more reliable one [14], which matches the precoded reliability bias of imbalanced neurons. Accordingly, we postulated that balanced neurons serve as a separation encoding basis in neural circuits since they are the counterparts of each other. In short, the balanced and imbalanced neurons have the potential to encode the spatial disparity of visual and vestibular cues in a reliability-based manner. We assumed that the ∆*θ*_mul_ distributions are identical in all directions; thus, only disparity coding was considered in this study, while specific directions were omitted. Next, we aimed to prove this hypothesis by a computational model that the response ratio, for either balanced or imbalanced neurons, is the dynamic origin that determines whether the MST-d neuron is a multisensory integration or separation encoder.

### 2.2. Continuous Attractor Neural Network (CANN) Modeling

**Figure 3:**
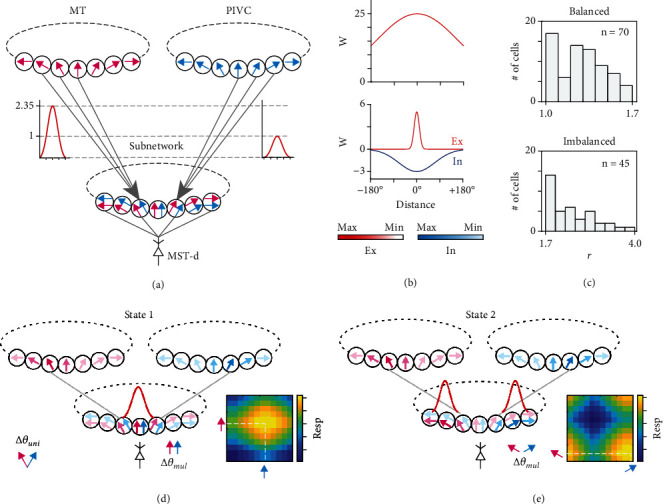
Multipartite CANN model structure and interpretation of preference. (a) The model structure. Each neuron stands for a specific direction preference. The synaptic connection weights are in proportion with the experimental observations. (b) Parameters of the model. Top: synaptic inputs to the MT and PIVC from preceding areas. The input weight declines with the topological distance of neurons. Bottom: lateral connection in a Mexican hat shape. (c) Synaptic strength ratio, determined from the data observed, is introduced in the model connection from the MT and PIVC to MST-d without specifying the visual or vestibular origin. (d) One state in the bifurcation system in the subnetwork, given fixed unisensory input directions. In this state, the subnetwork forms one coactivated region that assigns aligned preferences in the multisensory condition for the MST-d neuron downstream. The side panel shows a simulation trial in which the MST-d neuronal response achieves a maximum value when receiving congruent inputs. (e) The other state in the bifurcation system in the subnetwork. In this state, the subnetwork forms two independent regions that assign unaligned preferences in the multisensory condition. The side panel shows a simulation trial in which the MST-d neuronal response achieves a maximum value when receiving nearly opposite inputs.

To test our hypothesis that the response ratio for balanced or imbalanced neurons can determine neuronal encoding function, we constructed a multipartite cortical circuit model as a continuous attractor neural network (CANN, Figure 3(a); modified from [25], see Methods for details). The model simulated hierarchical sensory processing composed of three neural networks, two of which were the unisensory middle temporal region (MT) and parietal-insular vestibular cortex (PIVC). We assumed the third network to be a multisensory subnetwork that received the population outputs from the MT and PIVC regions and further determined the multisensory preference of MST-d neurons downstream. In other words, the CANN model simulated multisensory preference formation in specific synaptic input conditions; thus, it is independent of real stimuli.

#### 2.2.1. Model Sketches

Each network is composed of 180 neurons, whose positions denote preferences and are topologically aligned across networks. Neuronal dynamics are featured by a rate-based model in which activity ranges from 0 to 1 [25]. The inputs to MT and PIVC are visual and vestibular signals, respectively, and were simulated by a Gaussian function that centers at position *θ*_vis_ or *θ*_ves_ and has a wide range (Figure 3(b) top panel). Once the inputs were received, the neurons in two unisensory layers showed group response “bumps” mainly due to the lateral connections in a Mexican hat shape (Figure 3(b) bottom panel), and the center of the bump was usually distorted from *θ*_vis_ or *θ*_ves_ by neuronal intrinsic noise.

Then, the response bumps were sent forward to the multisensory subnetwork. Notably, the response ratio in the data reflects the synaptic property because the inputs were applied with the same motion intensity. It is well acknowledged that synaptic input is the product of synaptic weight and the input firing rate. Since the input firing rate is normalized to 1, we assumed that the synaptic input is proportional to the synaptic weight; thus, the response ratio is interpreted as the synaptic weight ratio (more concisely referred to as the synaptic ratio).

Therefore, balanced and imbalanced neurons were simulated by adjusting the proportion of the forward connection weights from the MT and PIVC to the subnetwork. The simulations were repeated 1000 times, and the occurrence of the weight ratio value followed the balanced (Figure 3(c) top panel) or imbalanced ratio distribution in the data (Figure 3(c) bottom panel).

When the forward inputs from the MT and PIVC arrived at the subnetwork, they still carried the distorted displacement between *θ*_vis_ and *θ*_ves_ due to the topological alignment setting. The neurons in the subnetwork responded to the inputs and formed bumps due to lateral connections (with the same parameters as unisensory layers), and the dynamics of the subnetwork are characterized by bifurcation states. It is obvious that when the inputs are close in distance, the neurons are prone to form a common response bump and cooperate (Figure 3(d)). In contrast, when the inputs are distant, the neurons are prone to form two independent bumps and compete (Figure 3(e)). Due to noise interference, both states occur by chance given fixed *θ*_vis_ and *θ*_ves_; thus, the final bump pattern is bifurcated. When neuronal responses are saturated in rate-based dynamic, both states are stable due to the countereffect of excitatory and inhibitory components in lateral connections, and the bumps do not collapse unless the inputs are removed.

#### 2.2.2. Model Interpretation

The bifurcation state of the subnetwork explicitly interprets the encoding of integration and separation for downstream MST-d neurons. The multisensory subnetwork serves as the receptive field of MST-d neurons. When real-time inputs deviate from the bump locations on the subnetwork, the response of the MST-d neurons is lower, which parallels the multisensory response function shown in Figure 1(c) (exemplified in the side panels of Figures 3(d) and 3(e), see also Supplementary Figure S1). Group cooperation indeed results in sensory integration because the visual and vestibular inputs share the same receptive field. In this manner, the response of MST-d neurons indicates that they originate from the same source. In contrast, group competition results in sensory separation because the MST-d response represents visual and vestibular inputs with distinct receptive fields and thus distinct sources. The integration and separation are robustly encoded by measurable neuronal responses. Consistent with data analysis, we denoted the distance |*θ*_vis_ − *θ*_ves_| as unisensory disparity ∆*θ*_uni_, which is a hyperparameter in CANN simulations. Since MST-d neuronal preference is characterized by the subnetwork, the distance between the responding bumps on the subnetwork is denoted as ∆*θ*_mul_. When only a common bump exists, ∆*θ*_mul_ is defined as 0°.

#### 2.2.3. Simulation Results

**Figure 4:**
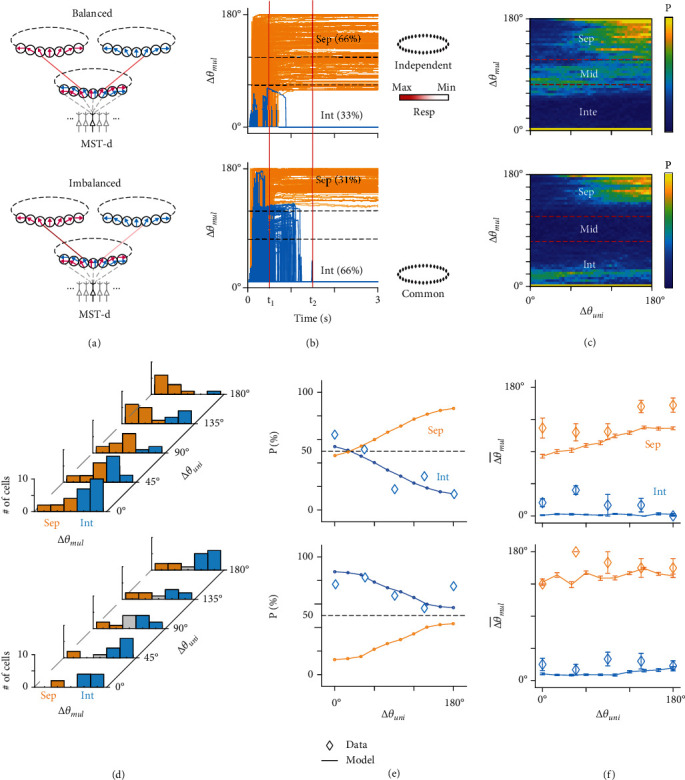
The CANN model simulates integration functions in the MST-d. (a) Schematics of balanced (top) and imbalanced (bottom) simulations in the CANN model. All other plots follow this arrangement. (b) Dynamic in the time domain. Five hundred trials are presented in each graph. The black dashed lines denote ∆*θ*_mul_ = 60° and ∆*θ*_mul_ = 120°. Side panels denote corresponding dominant response patterns in the subnetwork. (c) Dynamics in the space domain as a function of synaptic disparity (∆*θ*_uni_). ∆*θ*_mul_ is averaged from the time window *t*_1_ to *t*_2_, indicated by the red lines in (b). (d) Classified integration and separation-encoding neuron data in the joint distribution of Figure 2(f). (e) Probability of integration encoding (*p*_int_) as a function of ∆*θ*_uni_. *p*_sep_ = 1 − *p*_int_. Data: diamonds. CANN simulation: dotted curves. (f) Mean ∆*θ*_mul_ of integration-encoding as well as separation-encoding neurons (data: diamonds; CANN simulation: lines). The error bar denotes the standard error.

By introducing balanced and imbalanced forward ratios (Figure 4(a)), we first investigated the dynamic property in the time domain. Inputs were applied at *t* = 0 ms and maintained until the end of the trial (*t* = 3000 ms). Figure 4(b) shows that ∆*θ*_mul_ usually became stable after 1500 ms (arbitrary unit). In the balanced group, the majority of simulated neurons (658/1000 ≈ 66%) presented two independent response regions in the subnetwork, and the corresponding ∆*θ*_mul_ ranged from 60° to 180° in the end (Figure 4(b) top panel). However, in the imbalanced group, the majority (664/1000 ≈ 66%) had one common response region in the subnetwork, where ∆*θ*_mul_ = 0° in the end (Figure 4(b) bottom panel). Consistent with the data analysis, we chose the time window of 500 ms (*t*_1_) to 1500 ms (*t*_2_) and computed the mean ∆*θ*_mul_ in this window. Figure 4(c) shows that when MST-d neurons encode integration by a common response region, the mean ∆*θ*_mul_ does not necessarily decrease to 0° in the time window, especially for neurons in the imbalanced group. This validated that integration is encoded not only by neurons with ∆*θ*_mul_ = 0° but also by neurons with ∆*θ*_multi_ approximately 45°. Conversely, separation is encoded by balanced neurons with ∆*θ*_mul_ ∈ [60°, 180°] and imbalanced neurons with ∆*θ*_mul_ ∈ [120°, 180°].

Next, we correlated the encoding functions from CANN simulations to physiological data. As mentioned above, experiments involved a 45° range for each recorded direction; thus, the neurons with ∆*θ*_mul_ implied the range [∆*θ*_mul_ − 22.5°, ∆*θ*_mul_ + 22.5°]. We concluded that balanced neurons with ∆*θ*_mul_ ∈ {90°, 135°, 180°} encode separation, which includes a spatial range [67.5°, 180°] that is close to CANN predictions. On the contrary, balanced neurons with ∆*θ*_mul_ ∈ {0°, 45°} encode integration. For imbalanced neurons, those with ∆*θ*_mul_ ∈ {135°, 180°} encode separation, while those with ∆*θ*_mul_ ∈ {0°, 45°} encode integration. Imbalanced neurons with ∆*θ*_mul_ = 90° are reasonably omitted because they are both rare in CANN prediction and data (Figure 4(d) bottom panel, the 4 neurons with ∆*θ*_uni_ = 90° and ∆*θ*_mul_ = 90° were found to correlate more with balanced neurons with mean *r* = 1.84, which is significantly smaller than that of other imbalanced neurons, *p* = 0.021, *n*_1_ = 11, *n*_2_ = 34, two-sample *t*-test).

Figure 4(e) demonstrates the integration-encoding probability (*p*_int_) of MST-d neurons as a function of ∆*θ*_uni_^pref^ (hereafter briefed as integration function). *p*_int_ denotes *n*_int_/(*n*_int_ + *n*_sep_) at each ∆*θ*_uni_^pref^, where *n*_int_ is the integration-encoding neuron count and *n*_sep_ is the separation neuron count in the balanced or imbalanced group at each ∆*θ*_uni_. In the CANN model, *p*_int_ denotes *n*′_int_/(*n*′_int_ + *n*′_sep_), where *n*′_int_ is the number of trials that form one response region in the end and *n*′_sep_ forms two response regions in the end. The total trial count (*n*′_int_ + *n*′_sep_) is 1000 at each ∆*θ*_uni_. The CANN model fitted well with the experimental data. Both the data and simulations showed that the integration-encoding probability decreased with increasing synaptic disparity (∆*θ*_uni_). Accordingly, the separation-encoding probability increased. In general, the balanced neurons were biased to encode separation, while imbalanced neurons were biased to encode integration. Figure 4(f) also demonstrates the mean ∆*θ*_mul_^pref^ (${\bar{∆\theta }}_{\text{mul}}^{\text{pref}}$) conducted from integration or separation encoding neurons individually. CANN prediction in both balanced and imbalanced conditions reproduced data distributions.

In conclusion, the model simulated hierarchical processing from unisensory to multisensory regions and validated that MST-d neurons receiving balanced synaptic inputs generally encode sensory separation, while those receiving imbalanced synaptic inputs generally encode sensory integration. Jointly, the functional distinction enables MST-d balanced and imbalanced neurons to be effective bases for multisensory encoding. This proved our early hypothesis raised from experimental analysis that the balance level of synaptic coupling strengths from MT and PIVC input to MST-d neurons may be the key mechanism in driving individual MST-d neurons to be congruent neurons for integration or opposite neurons for segregation by a self-organization process.

#### 2.2.4. Dynamic Modulations from the Synaptic Ratio and Intrinsic Noise

We further investigated the role of neuronal intrinsic noise by altering noise intensities in the CANN model. Different noise intensities were simulated by Gaussian noise with 0 means and different standard deviations (*σ*_noise_). In each noise condition, we simulated the neuronal integration probability *p*_int_ with synaptic ratios of 1.0, 1.8, 2.2, and 2.6. Surprisingly, we found that neuronal intrinsic noise not only determined how distinct the encoding functions were but also altered the effective encoding bases themselves.

**Figure 5:**
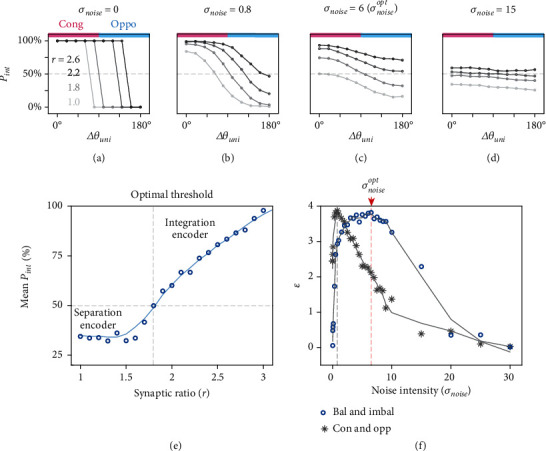
Dynamic modulation by the synaptic ratio and noise. (a–d) The integration function is modulated by both the synaptic ratio (from 1.0 to 2.6) and noise (from 0 to 15). (e) Modulation of the synaptic ratio determines neuronal function. The vertical dashed line shows the ratio that optimizes the threshold (*P*_int_ = 50%) between integration and separation. (f) Inference efficiency (*ε*) is modulated by the noise level (*σ*). The gray vertical dashed line shows the optimal noise level for congruent and opposite neurons as encoding bases (asterisks), and the red vertical dashed line shows the optimal noise level for balanced and imbalanced neurons as encoding bases (circles). *ε* on the *y*-axis is presented in arbitrary units based on a modified form of Kullback–Leibler divergence.

We first simulated a noise-free condition (*σ*_noise_ = 0, Figure 5(a)). In this condition, it was obvious that the model MST-d neurons encoded the inputs based on a fixed boundary (criterion) [30], and the boundary increased with the synaptic ratio (the criterion approximately 70°, 90°, 130°, and 150° as the ratio increased from 1 to 2.6). If the synaptic disparity range was in the boundary, the neuron robustly encoded integration; otherwise, it encoded separation. At the computational level, it is commonly acknowledged that effective encoding requires distinct neuronal responses. In this condition, effective encoding obviously relies on neurons preferring congruent or opposite stimuli, which is the congruent or opposite neurons in [1, 13]. When real inputs have a low degree of disparity, congruent neurons respond actively to infer integration, while opposite neurons remain inactive, and the individual can infer integration based on the response of congruent neurons and vice versa. Without loss of generality, we only discuss the encoding of disparity, while absolute direction is omitted.

With increasing noise levels (Figure 5(b)), the integration functions of different ratios became increasingly distinct from each other across the range of ∆*θ*_uni_ values, and the boundary in each function was blurred. At the noise level that fitted the data best, effective encoding was produced by balanced and imbalanced groups as computational bases (Figure 5(c)), which was the case we demonstrated above. In this case, integration was effectively inferred by a higher group response from imbalanced neurons and separation from balanced neurons. In other words, neuronal intrinsic noise transferred the encoding bases from congruent and opposite neurons to balanced and imbalanced neurons, where the synaptic ratio played a dominant role to discriminate the functions. The encoding of congruent and opposite neurons became less effective because their responses were usually similar under noisy conditions, shown as flatter integration functions across the range of ∆*θ*_uni_^pref^ values.

At a high noise level (*σ*_noise_ = 15, Figure 5(d)), all integration functions approximated 50% since randomization was dominant, and the modulation from the synaptic ratio also deteriorated. Thus, neither encoding by congruent and opposite neurons nor encoding by balanced and imbalanced neurons was efficient.

These results suggested that the synaptic ratio and intrinsic noise are both keys to neuronal multisensory computation. To quantify the modulation of the synaptic ratio, the integration functions were averaged as ${\bar{p}}_{\mathrm{int}}$ at the best-fit noise level (${\bar{p}}_{\mathrm{int}}=\sum_{i=1}^{n}p_{\mathrm{int},∆\theta_{\text{uni}}^{i}}/n$). With the increasing synaptic ratio, MST-d neurons were automatically classified as integration or separation encoders by a threshold of 1.8 (Figure 5(e)). When the ratio was less than 1.8, neurons generally served as separation encoders on average. When the ratio was greater than 1.8, they generally became integration encoders. Thus, the encoding function of MST-d neurons was dynamically determined by the synaptic ratio. Notably, the threshold we chose to classify the data (1.7) in physiological analysis was close to this optimal threshold.

Moreover, we propose that the typical balanced (*r* = 1.28) and imbalanced (*r* = 2.35) encoding was enhanced by the stochastic resonance (SR) mechanism (Figure 5(f)). SR refers to the situation in which the existing noise improves the input and output signal-to-noise ratio [31–33]. In multisensory encoding, the effect of SR was interpreted as inference efficiency (*ε*) measured by the revised Kullback–Leibler divergence between the integration functions of two encoders (see Methods). Intuitively, this can be interpreted as more effective encoding if the function curves deviate more from each other, allowing clearer representations.

The model predicted that maximum efficiency was achieved when *σ*_noise_ = 6.5 (≈0.26 times the peak amplitude of the model external input), which was close to the optimal noise level (*σ*_noise_^opt^) of 6 in our model. This indicated that real MST-d neurons achieved near-optimal encoding in the noisy physiological environment. Figure 5(f) also demonstrates the rescaled efficiency of congruent and opposite encoding (see Methods). In this case, the efficiency was maximized when *σ*_noise_=0.8 (≈0.03 times the peak amplitude of the model external input), suggesting that encoding by congruent and opposite neurons was most efficient in the low-noise condition. Nevertheless, encoding by balanced and imbalanced neurons was more efficient over a wide range of noise intensities. In the predicted physiological noise condition (*σ*_best‐fit_), the efficiency of balanced-and-imbalanced encoding was 185% compared to that of congruent-and-opposite encoding (in Supplementary Figure S3, the model results with intrinsic noise following a uniform distribution are also presented).

#### 2.2.5. Dynamic Modulation from Lateral Connections

**Figure 6:**
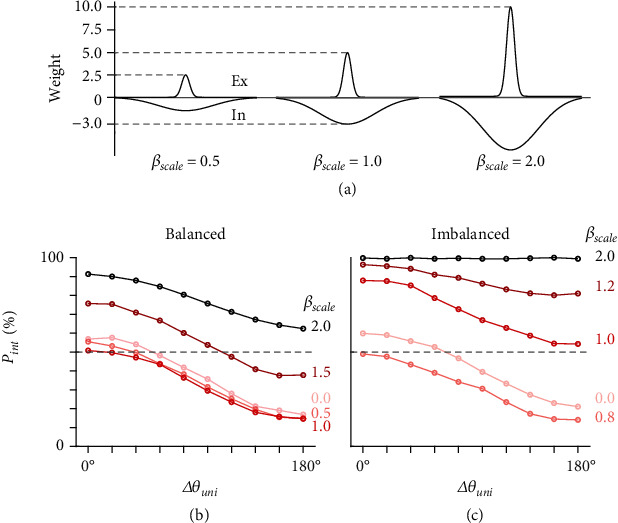
Dynamic modulation by lateral connections in the multisensory subnetwork. (a) Schematics of lateral-connection scaling by *β*_scale_, where *w*′_ex_ = *β*_scale_∙*w*_ex_ and *w*′_in_ = *β*_scale_∙*w*_in_. An example in which *β*_scale_ = 1.0 correlates with the best-fit case. (b) CANN simulated encoding functions of balanced neurons are modulated by lateral connection weights. (c) The same content as in (b), but for imbalanced neurons. *β*_scale_ is slightly different from (b) to moderately differentiate the curves.

Based on the CANN model results, we propose that balanced and imbalanced neurons comprise effective encoding with the help of intrinsic noise. We further investigated the role of lateral connections in the subnetwork. Potentially, these connections have a critical role in the encoding functions because the countereffect from excitatory and inhibitory connections is the main cause of the bifurcation states. We introduced a scale factor (*β*_scale_) to both excitatory and inhibitory components to increase or decrease the lateral connection weights (Figure 6(a)), while other parameters held the same weights. The best-fit CANN model refers to *β*_scale_ = 1.0. Figure 6(b) presents the integration-encoding functions in balanced simulations given different *β*_scale_ values. Separation encoding was robust when lateral weights decreased by half (*β*_scale_ = 0.5). Notably, the balanced neurons still encoded separation even when lateral connections were removed (*β*_scale_ = 0), although the integration probability was slightly larger. This is possibly due to the removal of inhibitory components, which is critical in competition among regions. When lateral weights increased (*β*_scale_ ∈ {1.5,2}), the function of balanced neurons was shifted to encode integration. For imbalanced neurons (Figure 6(c)), neuronal function was augmented to encode separation when lateral weights decreased (*β*_scale_ ∈ {0.75,0}), but the function to encode integration became more robust (*p*_int_ ~ 1) when lateral weights increased (*β*_scale_ ∈ {1.25,2}).

In the best-fit model (*β*_scale_ = 1), the total lateral input to one neuron in the subnetwork was nearly one-fifth of the total forward inputs. Although the lateral inputs appeared to be subordinate, these results indicated that lateral connections in the subnetwork are also critical to the functional distinction. In conclusion, effective encoding must meet certain requirements of the synaptic ratio (ratio between forward weights), presence of noise, and adequate lateral weights on the multisensory layer. These three factors comprise the neural mechanism of MST-d dynamic encoding.

### 2.3. Inference Decision Arising from MST-d Populational Network

The analysis above demonstrated the hierarchical mechanism of computing function of individual MST-d neurons in multisensory integration and separation. In this section, we took a further step along the cortical hierarchy and investigated the underlying algorithm of multisensory decision based on the existing of integrators and separators in the circuit. Specifically, we sought to resolve the debate about whether the high-level cortex performs Bayesian decision or non-Bayesian decision, such as those with deterministic strategies [30].

By simulating a bioplausible decision-making process, we prove that a single MST-d neuron utilizes fixed-criterion (FC) strategy, which makes deterministic inferences based on explicit boundaries (non-Bayesian). However, at the population level, the MST-d neuron group may compute causal inference in a reliability-based Bayesian strategy, which takes each MST neuronal response as a sampling of prior distribution to calculate the posterior probability of the cue origin state accordingly.

#### 2.3.1. Single-Neuron Level

The causal inference here denoted the binary judgment of whether the observed visual and vestibular inputs (*x*_vis_ and *x*_ves_) are attributable to one common cause (*C* = 1) or two separate causes (*C* = 2).

(6) $$\begin{array}{c} P\left(C=1\right)+P\left(C=2\right)=1. \end{array}$$

**Figure 7:**
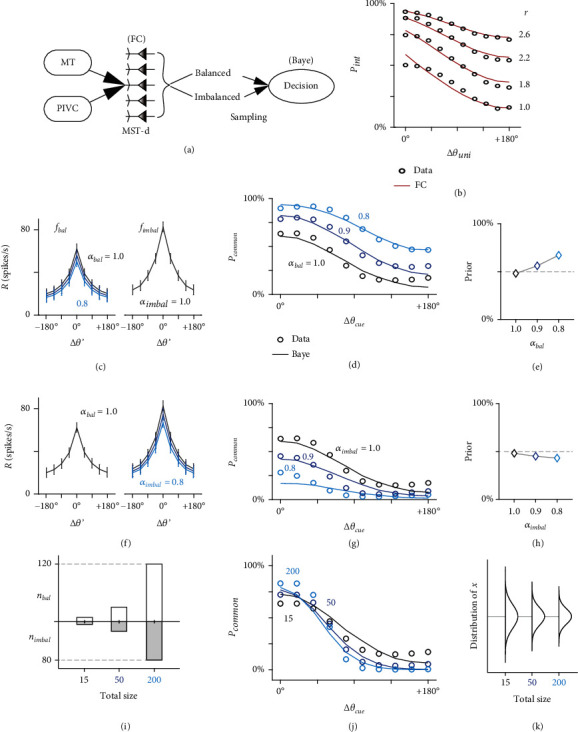
The fixed-criterion and Bayesian inference strategies emerge on different levels of the cortical hierarchy. (a) Schematics of cortical inference from single MST-d neurons implementing a fixed-criterion (FC) strategy to neurons in the MST-d group implementing a Bayesian strategy. (b) The FC strategy fitted with the neuronal integration functions with different synaptic ratios. (c) Multisensory response functions of balanced (*f*_bal_, left) and imbalanced (*f*_imbal_, right) neurons. The response decrement of balanced neurons is simulated by *R*′_bal_ = *α*_bal_∙*R*_bal_, where *α*_bal_ = 1, 0.9, or 0.8. *α*_imbal_ holds at 1. (d) Decisions simulated from the sampling process with different balanced response scales (circles) and Bayesian fitting (curves) with different prior probabilities while other parameters are fixed. The decision was about whether to report a common source or different sources, and the results are shown as probability (*p*_common_). The color matches the scaling condition in (c). (e) The best-fit prior in Bayesian fitting, shown as a function of *α*_*bal*_. (f–h) Same as (c–e), but for imbalanced neuron scaling by *α*_imbal_. The parameters in Bayesian fitting are the same as those in (d), except for the prior. (i) Schematics of different sample sizes. The top panels show the balanced sampling size, and the bottom panels show the imbalanced sampling size. A proportion of 3 : 2 was maintained. (j) Decision from sampling with different total sizes, as shown in (i) (circles), and Bayesian fitting with different measurement distributions (curves). The prior is fixed at 0.51. (k) The distribution relationship used in the fitting of (j).

We first focused on the inference performed by a single neuron. Under noise-free conditions, we verified in the last section that MST-d neurons adopt an FC strategy, which means that the neurons infer a common cause (*C* = 1) if the two measurements are closer than a fixed boundary *κ* (|*x*_vis_ − *x*_ves_| < *κ*) and infer two separate causes (*C* = 2) otherwise. The strategy is deterministic because the boundary *κ* is explicit and determined [30]. However, the presence of neuronal intrinsic noise (*ξ*) blurs the measured disparity. To test whether MST-d neurons still follow the FC strategy in the noisy condition, we simulated the noisy FC strategy as |*x*_vis_ − *x*_ves_| + *ξ* and fitted the integration decision from this strategy with the neuronal integration functions (see Methods). The parameter was boundary *κ*, which varied across functions, and the noise level was *ξ*.  Figure 7(b) demonstrates that the noisy FC strategy closely reproduced integration functions in the case of different synaptic ratios (data here refer to CANN functions for higher ∆*θ*_uni_ resolution). This suggested that in the noisy condition, each MST-d neuron still performed deterministic inference with fixed and explicit boundaries, and the boundary varied among neurons due to different synaptic ratios.

#### 2.3.2. Population Level

At the MST-d population level, we postulated that a new strategy emerges due to the pooling of various boundaries. Since the decisions are decoded from responses in neural circuits, we first characterized the MST-d neuronal multisensory response properties based on the recorded data. The multisensory response was characterized as a function of both preferred disparity and real cue disparity (*f*_bal_ and *f*_imbal_, Figure 7(c)). Both *f*_bal_ and *f*_imbal_ were obtained by averaging the multisensory responses relative to the preferred condition in real data (see Supplementary Figure S2 for methods), and they characterized the real-time balanced or imbalanced neuronal response to hypothetical cues as *R* = *f*(∆*θ*′), where ∆*θ*′ = |∆*θ*_mul_−∆*θ*_cue_|. For example, if ∆*θ*_cue_ is 30°, the neurons for which ∆*θ*_mul_=30° exhibit a maximal response because *f*(|∆*θ*_mul_−∆*θ*_cue_|) = *f*(∆*θ*′ = 0°) = *R*_multi_^max^. However, the neurons for which ∆*θ*_mul_=180° have low responses because *f*(∆*θ*′ = 150°). We simulated ∆*θ*_cue_ from 0° to 180° in the horizontal plane, which is the same as the experimental conditions.

Second, we adopted a probabilistic Monte Carlo sampling process of neuronal responses in balanced and imbalanced groups individually. The response sampling is widely observed along the cortical hierarchy [34, 35]. Here, the sampling of MST-d neuronal responses simulated that MST-d neurons were randomly activated by fixed ∆*θ*_cue_ in noisy physiological condition, and each neuronal response served as a prior sample of the cues based on the inherent tuning property. To seek a minimal requirement to realize flexible decisions, we sampled 15 neurons from MST-d group, 9 of which were balanced neurons and 6 of which were imbalanced neurons. The proportion followed data observations (*n*_bal_ : *n*_imbal_ = 70 : 45 ≈ 3 : 2).

Next, the sampled responses were summed as balanced or imbalanced group responses and sent to a decision neuron, which compared the group response amplitude to reach a binary decision (the limited sample size is plausible because it is little likely that responses of all MST-d neurons are sent to a common neuron downstream). Since we proved that imbalanced neurons are integration encoding bases and balanced neurons are separation encoding bases, the resultant decision was to integrate the senses and report common source if imbalanced groups had higher responses or to separate the senses and report different sources otherwise. At each ∆*θ*_cue_, we simulated such binary decision-making for 100,000 times to obtain the averaged decision probability of reporting a common source (*p*_common_) as a function of external cue disparity (Figure 7(d)).

It is crucial to note that balanced and imbalanced neurons are actually reliability-based. Previous works proved that imbalanced neurons are more sensitive to reliability changes in the dominant cue but less sensitive to those in the subordinate cue [36]. On the other hand, balanced neurons have equal sensitivity to both cue reliabilities. Thus, it is reasonable to expect that when one cue is unreliable, the response of the balanced neurons is weaker, while the imbalanced neurons that prefer the other cue maintain the response level (we assume each decision is made with imbalanced neurons with the same cue dominance). The response change was simulated by amplitude scaling (*R*′ = *α*∙*R*), where *α* is the scale factor. In this situation, the amplitude change was expressed as *α*_bal_ < 1 and *α*_imbal_ = 1. We simulated a mild decrease to balanced responses as *α*_bal_ ∈ {0.9, 0.8}, and we presented that the probability of reporting a common source (*p*_common_) significantly shifted toward 100% (Figure 7(d), circles, from black to light blue), which means that the decision was prone to integrate cues when a specific cue was unreliable.

To specify the strategy during such decisions, we simulated classical Bayesian optimal inference [20] (see Methods). The Bayesian strategy differs substantially from the FC strategy because it computes the posterior probability of *C* = 1 or *C* = 2 based on sampled measurements *x*_vis_ and *x*_ves_ and prior *p*(*C* = 1). We considered the Bayesian strategy here because the prior may be computed by pooling the fixed boundaries during the decision. In the simulation, the prior was set as a parameter that varied across the decision functions.

As the curves in Figure 7(d) demonstrated, the Bayesian strategy approximated well with decisions from the sampling process. Notably, the case when *α*_bal_ = 1 and *α*_imbal_ = 1 matched the real neuronal recording when both cues represented the same motion. In this case, the best-fit prior was 0.48, which was close to the flat prior (0.5). This result suggested that the inference in the adult brain assumes a fair prior when both cues are equally reliable. Furthermore, when the reduced reliability of one cue was projected to the weaker response of balanced neurons, the prior increased accordingly (Figure 7(e)) and resulted in a higher probability of reporting a common source.

Next, we considered the other case in which the dominant cue is unreliable. Consequently, the imbalanced neuron response decreased more than the balanced neurons. It is obvious that when both responses are decreased proportionally, the decision is the same as that when *α*_bal_ = 1 and *α*_imbal_ = 1. Thus, we simulated the case in which *α*_bal_ = 1 and *α*_imbal_ ∈ {0.9, 0.8} instead (Figure 7(f)). We presented data in Figure 7(g) that decisions from the sampling process were biased to different sources in this case. The Bayesian strategy consistently approximated the decisions with decreasing prior (Figure 7(h)). In conclusion, we proved that the Bayesian strategy naturally emerged from the encoding of balanced and imbalanced encoding bases. The two bases linked disparity coding with cue reliability in the form of prior computation and produced flexible decisions that varied accordingly with cue reliability. In other words, the fMCS model provided a physical base to derive multisensory decision by the posterior probability of integration based on the response samples in MST-d.

Finally, we demonstrated the Bayesian interpretation of sample size (total size = *n*′_bal_ + *n*′_imbal_, Figure 7(i)). Note that the sample size in this section is independent of the sample size in experimental recordings. The variation of sample size could result from inherent synaptic connection from MST-d neurons to the specific decision neuron or from different intensities of external cues, where strong intensity activates more MST-d neurons, and more samples participate in a decision trial therewith. With an increasing total sample size, the decision functions showed steeper gradients (Figure 7(j)), indicating that decisions were made with more confidence. In other words, accumulating evidence is represented by more neurons participating in the decision. This is consistent with behavioral conclusions that the more evidence accessible, the more confidence is associated with the decision [37, 38]. The decision functions were nicely fitted by a Bayesian strategy with a narrower probability distribution, while other parameters were fixed (Figure 7(k), *p*(*x*_vis_|*s*) and *p*(*x*_ves_|*s*), where *s* denotes the sensory source and *x* the sensory measurements; prior probability = 0.51 in these simulations; see Methods for details). The narrowing distribution in Bayesian fitting matched the statistical rules that standard error decreases with increasing sample size (standard $\text{error}=\sigma /\sqrt{n}$, where *σ* is the standard deviation of sensory measurements and *n* is the sample size). These results strongly proved the emergence of a probabilistic Bayesian strategy from coding in the balanced and imbalanced groups of neurons.

Previous works argued that both FC and Bayesian strategies are likely to underlie brain inference, and we validated that the two strategies function on different levels. Crucially, a Bayesian decision cannot be reduced to the summation of individual neurons that perform FC strategy only. This is because each neuron carries a fixed prior representation, but the change in the prior is computed by pooling neurons with different synaptic features during a decision. Such group coding allows the realization of a flexible probabilistic decision that is biased to integrate the inputs from different modalities when one of them is not reliable and separate them when both of them are reliable. This section proposed the Bayesian-decision emergence by varying neuronal response. We also presented in Supplementary Figure S4 that change of proportion between balanced and imbalanced neurons also produced Bayesian-like decision, where the prior has more drastic bias. The change of proportion could result from various neuronal activation threshold in physiological condition. The proportion change added to the multisensory inference flexibility in real brain.

## 3. Discussion

This study is aimed at revealing the neural encoding mechanism of multisensory causal inference in the MST-d. The novel focus of this paper is to prove computationally that the balance level of synaptic coupling strengths from MT and PIVC inputs to MST-d neurons may be the key mechanism in driving the MST-d neurons to be congruent encoders for integration or opposite/intermediate encoders for segregation by a self-organization process. The computational results also demonstrated another novel mechanism that produces the maximum coding capacity by stochastic resonance with the optimal intensity of intrinsic noise. Based on these mechanisms, we further demonstrated that each MST-d multisensory neuron implements a non-Bayesian FC strategy, but the prior emerges by pooling diversified criteria. Moreover, MST-d neuron populations implement a probabilistic Bayesian strategy. Therefore, this study established a computational framework that the feedforward inputs from early pathways play a key role in determining the emergence of congruent and opposite neurons in multisensory decoding, and sensory motion discrimination requires both Bayesian and non-Bayesian strategies, each serving at the single-neuron or populational level.

### 3.1. Synaptic Coupling Generates a Novel Set of Neural Bases for Multisensory Encoding

Previous works generally assumed that unisensory congruent neurons perform sensory integration, while opposite neurons perform sensory separation; theoretical works verified this assumption through vector computation [1, 13]. However, there are also works demonstrating that congruent neurons can perform separation, and opposite neurons can perform integration [39].

In this study, we first pointed out that neuronal preference in multisensory conditions is usually different from that in unisensory conditions. Therefore, implementing multisensory coding by unisensory distributions generally led to confusion. Second, we proposed the novel encoding bases of balanced and imbalanced neurons, whose encoding properties originates from inherent synaptic features but not unisensory features. In this case, the congruent and opposite neurons are not tightly correlated with the balanced and imbalanced categories (Supplementary Figure S5). The CANN model further demonstrated a neuronal encoding mechanism based on balanced and imbalanced neuronal bases, which is nearly twice as efficient as the mechanism based on congruent and opposite bases (Figure 5(f)). Notably, our results solved three outstanding problems. (1) Unisensory intermediate neurons are usually omitted in functional analysis. Here, we confirmed that these neurons also participated in dynamic multisensory encoding, and they are equally functional as unisensory congruent and opposite neurons. (2) We confirmed the finding from Rideaux et al. through physiological analysis and revealed that multisensory encoding is probabilistic. (3) The vector computation proposed by Zhang et al. does not cover the neuronal functions when ∆*θ*_mul_ = 90°. We specified the role of these neurons as separation encoding, which explained behavioral observations that separation estimation generally has less estimated error than integration [20, 40]. In our theory, the separation encoding basis is the balanced neuron group, which has more neurons encoding a wider range of disparity (from 60° to 180°). Therefore, separation encoding has higher resolution in comparison because the disparity is detected by more neurons.

In our theory, causal inference emerges from the sensory processing hierarchy from unisensory to multisensory cortical areas, which has been verified by fMRI studies [21]. As the means of connection of this hierarchy, the synaptic coupling mechanism explains the finding in evolutionary biology that multisensory computation emerges postnatally with the development of synaptic connections [41]. In the superior colliculus (SC) of the cat, multisensory neurons are initially unable to integrate combinations of sensory cues to produce significant enhancement or depression of responses [42], but the ability to integrate cross-modality inputs gradually increases with postnatal experience as cortical SC synapses are modified following Hebbian rules [43]. Furthermore, the findings of the present study are in line with functional conclusions that the spatial reference frame of the MST-d varies as a function of the relative strength of visual and vestibular inputs when different modalities are combined [44].

### 3.2. Model Interpretation of the Subnetwork

We propose that the subnetwork is necessary for multisensory attractor computation, while the recorded MST-d neurons mainly serve to report the inference. There are a few plausible explanations for this subnetwork identity. First, this network belongs to an anatomical subregion in MST-d. In this case, the responding units involved in separation encoding may be recorded as singularly tuned neurons because they only respond to a single input, while in integration encoding, the responding units may be considered multisensory neurons with aligned preferences. This hypothesis will be further substantiated if structural organization in MST-d is revealed in the future. Another potential identity is the ventral intraparietal area (VIP). The VIP meets three requirements crucial to attractor computation: (1) the VIP is anatomically connected with the PIVC [45] and reciprocally connected with the MT [46]; thus, in the multisensory condition, the response of the VIP may be fed back to preceding cortical areas to change the receptive field property. (2) Almost half of the neurons in the VIP are dominated by congruent visual and vestibular preferences [47]. This is crucial to the multisensory attractor computation because it passes ∆*θ*_uni_ from unisensory areas to a common level and the disparity feature is preserved. (3) VIP neurons have a larger activity correlation than MST-d neurons measured by correlated noise [47], which indicates that VIP neurons process multisensory signals more intensively than other neurons. Although it seems inappropriate to assume a whole network computation for each recorded MST-d neuron, the majority of neurons in this subnetwork are redundant and can be released from the circuit after synaptic modification, and only specific neurons carrying multisensory direction preference are preserved. It is also possible that the hypothetical subnetwork stands for an undiscovered type of multisensory synaptic attractor learning rule, which requires further work.

### 3.3. Probabilistic Bases in Group Encoding Are the Key to Flexible Probabilistic Decision-Making

MST-d neurons are predetermined as integration and separation encoders based on synaptic inputs. Due to noise, balanced and imbalanced groups have distinct functions in multisensory inference, which makes them ideal computational bases to maximize inference efficiency. Although we consider each MST-d neuron an essential encoding component in a decision, our theory still supports the idea that MST-d group encoding is the foundation of multisensory discrimination [20]. Since the FC strategy applied by each MST-d neuron also stems from the group coding of the preceding unisensory areas, it seems that the complexity increases as downstream cortical neurons receive preceding inputs and produce outputs. Although the probabilistic decision substantially matches the behavioral results of similar tasks [35, 48–50], the psychophysical results show low resolution, with the threshold between integration and separation discrimination being approximately 47° (Figure 7(g)), whereas the threshold in behavioral tasks lies at 20°. Other mechanisms, such as top-down modulation or crosstalk between several multisensory areas, might be necessary to further improve the resolution [30].

While many works have focused on the direct correlation between one decision and a specific neural basis, our results indicated that real cognitive performance may not be able to be reduced to a corresponding smaller structure but may instead emerge naturally through a series of probability distributions of bases. We presented in Supplementary Figure S6 that a clear correlation between the causal scenario and neurons only makes rigid decision, while the distributed preference as we observed in data produces maximal inference efficiency. In conclusion, the brain functions as an integrated hierarchy, and neural firing in different cortical areas represents different features. In multisensory areas, it is likely that single neuron encodes multiple features that is either reliability-based or modality-based. The multidimensional encoding may serve as the key to produce flexible intelligence behavior.

### 3.4. Limitations of This Study and Suggestions for Potential Future Research

The physiological results must be interpreted with caution because of the limited sample size. Furthermore, the model used a simplified structure that excludes real neuronal synaptic dynamics, such as neural firing heterogeneity within and across the cortical area. The diversity of synapses may enrich the dynamics of integration and separation inference. Moreover, the model did not employ a synaptic learning process to transfer the characteristics of the response subnetwork to the receptive field property. Regarding possible future directions, further study is needed concerning receptive field modification to develop a complete attractor computation process. Additionally, further studies can test this synaptic modulation theory in other multisensory areas, such as the VIP, to specify the scale on which synaptic encoding modulation functions as a general mechanism.

## 4. Methods

### 4.1. Subjects and Surgery

Two male rhesus monkeys (*Macaca mulatta*) served as subjects. The general procedures followed in this study have been described previously [51, 52]. Each animal was outfitted with a circular molded plastic ring anchored to the skull with titanium T-bolts and dental acrylic. To monitor eye movements, a scleral search coil was implanted in each monkey. The Institutional Animal Care and Use Committee at Washington University approved all animal surgeries and experimental procedures, which were performed following National Institutes of Health guidelines. Animals were trained to fixate on a central target for fluid rewards using operant conditioning.

### 4.2. Vestibular and Visual Stimuli

A 6-degree-of-freedom motion platform (MOOG 6DOF2000E; Moog, East Aurora, NY) was used to passively translate the animals along one of eight directions in the horizontal plane, spaced 45° apart. A tangent screen was affixed to the front surface of the field coil frame, and visual stimuli were projected onto it by a three-chip digital light projector (Mirage 2000; Christie Digital Systems, Cypress, CA). The screen measured 60 × 60 cm and was mounted 30 cm in front of the monkey, thus subtending ~90° × 90°. The visual stimuli simulated translational movement along the same eight directions through a three-dimensional cloud of stars. Each “star” was a triangle that measured 0.15 cm × 0.15 cm; the cloud measured 100 cm wide by 100 cm tall by 40 cm deep and had a star density of 0.01 per cm^3^. To provide stereoscopic cues, the cloud was rendered as a red–green anaglyph and viewed through custom red–green goggles. The optic flow field contained naturalistic cues mimicking the translation of the observer in the horizontal plane, including motion parallax, size variations, and binocular disparity.

### 4.3. Electrophysiological Recordings

We recorded action potentials extracellularly from both hemispheres in each of the two monkeys. For each recording session, a tungsten microelectrode was passed through a transdural guide tube and advanced using a micromanipulator. An amplifier, an eight-pole bandpass filter (400–5000 Hz), and a dual voltage-time window discriminator (BAK Electronics, Mount Airy, MD) were used to isolate action potentials from single neurons. Action potential times and behavioral events were recorded with 1 ms accuracy by a computer. Eye coil signals were processed with a low-pass filter and sampled at 250 Hz.

Magnetic resonance imaging (MRI) scans and Caret software analyses along with physiological criteria were used to guide electrode penetration into the MST-d area [1]. Neurons were isolated while a large field of flickering dots was presented. In some experiments, we further advanced the electrode tip into the lower bank of the superior temporal sulcus to verify the presence of neurons with response characteristics typical of the MT [1]. Receptive field locations changed as expected across guide tube locations based on the known topography of the MT [1].

### 4.4. Experimental Protocol

We measured neural responses to eight heading directions evenly spaced every 45° in the horizontal plane. Neurons were tested under three experimental conditions. (1) In vestibular trials, the monkeys were required to maintain fixation on a central dot on an otherwise blank screen while being translated in one of the eight directions. (2) In visual trials, the monkeys were presented with optic flow simulating self-motion (in the same eight directions), while the platform remained stationary. (3) In bimodal trials, the monkeys experienced both translational motion and optic flow. We paired all eight vestibular headings with all eight visual headings for a total of 64 bimodal stimuli. Eight of these 64 combinations were strictly congruent, meaning that the visual and vestibular cues simulated the same heading. The remaining 56 cases had conflicting cue stimuli. This relative proportion of strictly congruent and conflicting stimuli was adopted purely to characterize the neuronal combination rule and was not intended to be ecologically valid. Each translation followed a Gaussian velocity profile. It had a duration of 2 s, an amplitude of 13 cm, a peak velocity of 30 cm/s, and a peak acceleration of 0.1 × *g* (981 cm/s^2^).

These three stimulus conditions were interleaved randomly along with blank trials, which included neither translation nor optic flow. Ideally, five repetitions of each unique stimulus were collected for a total of 405 trials. Experiments with fewer than three repetitions were excluded from the analysis. When isolation remained satisfactory, we ran additional blocks of trials with the coherence of the visual stimulus reduced to 50% and/or 25%. Motion coherence was lowered by randomly relocating a percentage of the dots on every subsequent video frame. For example, we randomly selected one-quarter of the dots in every frame at 25% coherence and updated their positions to new positions consistent with the simulated motion, while the other three-quarters of the dots were plotted at new, random locations within the 3D cloud. Each block of trials consisted of both unimodal and bimodal stimuli at the corresponding coherence level. When a cell was tested at multiple coherence levels, both unimodal vestibular tuning and unimodal visual tuning were independently assessed in each block.

Trials were initiated by displaying a 0.2° × 0.2° fixation target on the screen. The monkeys were required to fixate on the target for 200 ms before the stimulus was presented and to maintain fixation within a 3° × 3° window to receive a liquid reward. Trials in which the monkeys broke fixation were aborted and discarded.

### 4.5. Data Analysis

The neural responses were binned in 25 ms time windows. Mean neural responses were averaged from 5 trials, and the units of measurement were spikes per second. The outliers in the 5 trials were removed, and the mean response was averaged from the remaining 4 trials. Using MATLAB (MathWorks, Natick, MA), we chose the window of 625 ms to 1250 ms to select valid data. We considered a neuron to have discriminative tuning properties to one specific stimulus modality if the maximum response was 5 spikes/s more than the minimum response of the same curve. Tuning curve symmetry was not considered. Neurons that failed to meet this requirement for either the visual or the vestibular unisensory condition were considered unisensory-tuned neurons or poorly tuned neurons and removed from further analysis. Then, we computed the response ratio based on the visual and vestibular unisensory tuning curves of that neuron in the same time window. A threshold of 1.7 was chosen to discriminate between balanced and imbalanced data. We analyzed the tuning curve in the time window from 1000 ms to 1250 ms, which corresponds to the maximum motion speed and maximum neural response (data not shown). The window parameters were first scanned and then selected comprehensively to show the discrimination of the integration probability *P*_int_ between the balanced and imbalanced neurons and to be physiologically plausible.

The group Δ*θ* distribution was rectified by doubling the probability at 0° and 180°, while the probabilities in other directions remained the same. This procedure was followed because of the experimental protocol in which directions were binned in 45° intervals; a 0° preference encompassed preferences from -22.5° to +22.5°, and a 180° preference encompassed preferences from 167.5° to 202.5°. However, each of the other preference bins was represented twice because both sides were included (for example, Δ*θ* of 45° encompassed directions from 22.5° to 67.5° and from -22.5° to -67.5°). To align the widths of the probability bins, the data counted at 0° and 180° were included twice.

### 4.6. Continuous Attractor Neural Network Modeling

The model is composed of three identical neural networks with hierarchical structures, of which two simulate the unisensory middle temporal region (MT) and parietal-insular vestibular cortex (PIVC), while the third simulates a subnetwork. Each network is specified as a ring attractor with the same structure. We consider a network of *L* (180) neurons *i* ∈ [1, 2, ⋯, *L*] arranged along a ring topology, each representing a 2° direction range (preference) in the external world. Due to the circular structure, neuron *i* = *L* is a neighbor of neuron *i* = 1, and we defined a circular distance *d*(*i*, *j*) between neurons *i* and *j* as *d*(*i*, *j*) = min(|*i* − *j*|, *L* − |*i* − *j*|), which takes a value from 0°~180°.

Without loss of generality, we adopted a normalized rate-based model to describe the neural dynamics, which was sufficient for attractor computation. The activity of neuron *k* in all layers is described by the following equation.

(7) $$\begin{array}{c} \tau \frac{d}{dt}y_{k}\left(t\right)=-y_{k}\left(t\right)+S\left(I_{k,r}+I_{k,e}+I_{k,\text{noise}}\right). \end{array}$$

*τ* is the time scale, and *y*_*k*_(*t*) is the neuronal activity. *S*(*I*) is the sigmoid activation function.

(8) $$\begin{array}{c} S\left(I\right)=\frac{1}{1+\exp\left(w∙\left(I-\varphi \right)\right)}. \end{array}$$

*I*_*k*,*r*_ is the recurrent connection within each ring network.

(9) $$\begin{array}{c} I_{k,r}=\overset{L}{\underset{j=1}{\sum }}w_{j,k}∙y_{j}\left(t\right). \end{array}$$

The connection weights *w*_*j*,*k*_ follow the Mexican hat profile, which is identical across layers.

(10) $$\begin{array}{c} {\left.w_{j,k}\right|}_{j\neq k}=w_{\text{ex}}∙\exp\left(\frac{-d_{j,k}^{2}}{2\sigma_{\text{ex}}^{2}}\right)-w_{\text{in}}∙\exp\left(\frac{-d_{j,k}^{2}}{2\sigma_{\text{in}}^{2}}\right), \end{array}$$

where *w*_ex_ and *w*_in_ are excitatory and inhibitory components (*w*_ex_ > *w*_in_) and *σ*_ex_ and *σ*_in_ characterize the excitatory and inhibitory range (*σ*_ex_ < *σ*_in_). *d*_*j*,*k*_ denotes the topological distance from the *j*^th^ neuron to the *k*^th^ neuron. As unisensory layers, MT and PIVC receive corresponding visual and vestibular external inputs with range *σ* from proceeding areas, whose intensity decays with the relative distance (*d*) from the input center (*θ*_max,vis_ and *θ*_max,ves_).

(11) $$\begin{array}{c} I_{k,e}=w_{\text{external}}∙\exp\left(\frac{-d{\left(\theta_{\max},k\right)}^{2}}{2∙\sigma_{\text{external}}^{2}}\right), \end{array}$$

where *w*_external_ is the maximal input weight and *σ*_external_ is the input range. Once the inputs are applied, the neurons in the same layer interact with each other through lateral connections and eventually form the group response in a bump shape on the layer. Meanwhile, the responses of two unisensory areas are sent to the subnetwork by forward synaptic connections, which are topologically aligned such that one neuron receives maximally weighted input from the upstream neuron at the same position.

(12) $$\begin{array}{c} I_{k,e}^{\text{sub}}=\overset{L}{\underset{i=1}{\sum }}w^{MT}∙\exp\left(\frac{-{d_{i,k}}^{2}}{2∙\sigma_{\text{forward}}^{2}}\right)∙y_{i}^{\text{MT}}+\overset{L}{\underset{j=1}{\sum }}w^{\text{PIVC}}∙\exp\left(\frac{-{d_{j,k}}^{2}}{2∙\sigma_{\text{forward}}^{2}}\right)∙y_{j}^{\text{PIVC}}, \end{array}$$

where *w*^MT^ and *w*^PIVC^ are forward synaptic weights from the MT and PIVC regions to the multisensory subnetwork. The ratio between two weights simulates balanced and imbalanced neurons. *σ*_forward_ characterizes the forward input range. *d* denotes the topological distance from the *i*^th^ MT neuron or *j*^th^ PIVC neuron to the *k*^th^ subnetwork neuron, whose activity is *y*_*i*_^MT^ and *y*_*j*_^PIVC^ individually.

Finally, each neuron is independently subjected to Gaussian intrinsic noise except for the individual MST-d multisensory neuron downstream.

(13) $$\begin{array}{c} \;I_{k,\text{noise}}=\psi \left(t\right),\\ \langle \psi \left(t1\right)∙\psi \left(t2\right)\rangle =0,\\ \langle \psi {\left(t1\right)}^{2}\rangle =\sigma_{\text{noise}}^{2}. \end{array}$$

When inferring the integration or separation from the response of the subnetwork, the threshold is set as 0.15 to discriminate effective population response from an undetectable response or neural avalanche. The cases in which all neurons in the final state have responses lower than 0.15 or higher than 0.15 were excluded. The threshold was fixed and used only when inferring the integration and separation trials. The optimal values of the parameters are listed in Table 1.

### 4.7. Neuronal Response Curve Simulation

We supplemented direction interpretation with observations from the data by projecting the computation of the CANN network on the MST-d neuron by the leaky integrate-and-fire model. The input for this model is an external stimulus with different directions and the output the neuronal fire rate. For a given MST-d neuron along with its circuit, the input direction is fixed by the receptive field, and the input intensity decreases when the real motion direction is misaligned with the preference. The unisensory preference is fixed by the assigned location of two inputs on the receptive field.

(14) $$\begin{array}{c} ∆\theta_{\text{uni}}=\min\left[\left|\theta_{\max,\text{vis}}-\theta_{\max,\text{ves}}\right|,L-\left|\theta_{\max,\text{vis}}-\theta_{\max,\text{ves}}\right|\right]. \end{array}$$

In the unisensory condition, each simulated neuron receives preceding inputs from the aforementioned directions when real input directions *θ* ∈ [0°, 360°].

(15) $$\begin{array}{c} I_{k,e}^{\theta }=w_{\text{external}}^{\theta }∙\exp\left(\frac{-d{\left(\theta_{\max}^{\text{uni}},k\right)}^{2}}{2∙\sigma_{\text{external}}^{2}}\right), \end{array}$$

where *w*_external_ is the external input weight at *θ*_max_^uni^ (same as that in the CANN model), whose intensity decreases when the real input direction *θ* deviates from *θ*_max_^uni^. Thus, the group output *Y* from either the MT or PIVC is the summation of each neuronal response,

(16) $$\begin{array}{c} Y_{c}=\overset{L}{\underset{i=1}{\sum }}y_{i,c},c\text{ is MT or PIVC}, \end{array}$$

where *y*_*i*,*c*_ is the *i*^th^ neuronal response on layer *c*. The afferent input current *I*_sti_(*t*) is further specified as

(17) $$\begin{array}{c} I_{\text{sti}}\left(t\right)=\alpha Y_{c}-\text{thr}. \end{array}$$

*α* denotes the rescaling factor (limited in this section), and thr denotes the signal detection threshold. The membrane potential (*V*) of neurons in the `MST-d is derived from the differential equation

(18) $$\begin{array}{c} C\frac{dV}{dt}=-g_{l}\left(V\left(t\right)-V_{m}\right)+I_{\text{sti}}\left(t\right), \end{array}$$

where *V*_*m*_ is the resting potential (-65 mV) and *V*(*t*) denotes the membrane potential at time *t*.

In the multisensory condition, the subnetwork is set to update the preference after 30 ms, and both inputs are sent to the MST-d.

(19) $$\begin{array}{c} I_{k,e}^{\theta }=w_{\text{external}}^{\theta }∙\exp\left(\frac{-d{\left(\theta_{\max}^{\text{multi}},k\right)}^{2}}{2∙\sigma_{\text{external}}^{2}}\right),\;t>30\text{ ms},\\ C\frac{dv}{dt}=-g_{l}\left(v\left(t\right)-v_{m}\right)+I_{\text{sti}}^{\text{MT}}\left(t\right)+I_{\text{sti}}^{\text{PIVC}}\left(t\right). \end{array}$$

### 4.8. Computation of Inference Efficiency Shown in Figure 6

We measured the inference efficiency based on cross-entropy (Kullback–Leibler divergence) since we measured the distribution of integration probability (though not the probability distribution, which requires ∑*p*_int_(*x*) = 1). Cross-entropy achieves a maximum value if two distributions are identical; however, the computational principle requires the encoding bases to differentiate distributions. Thus, we denote the inference efficiency *ε* by negative cross-entropy. To balanced and imbalanced neuronal encoding,

(20) $$\begin{array}{c} \varepsilon =\overset{180{}^{\circ}}{\underset{∆\theta =0{}^{\circ}}{\sum }}\left[p\left(y_{\text{bal}}\right)\log_{2}p\left(y_{\text{imbal}}\right)-p\left(y_{\text{bal}}\right)\log_{2}p\left(y_{\text{bal}}\right)\right]. \end{array}$$

To congruent and opposite neuronal encoding,

(21) $$\begin{array}{c} \varepsilon^{\prime }=\overset{∆\theta_{0}+90{}^{\circ}}{\underset{∆\theta_{0}}{\sum }}\left[p\left(y_{\text{oppo}}\right)\log_{2}p\left(y_{\text{cong}}\right)-p\left(y_{\text{oppo}}\right)\log_{2}p\left(y_{\text{oppo}}\right)\right], \end{array}$$

where ∆*θ*_0_ is 0° in congruent neurons and 90° in opposite neurons. The congruence and opposite cases are set as mutually exclusive here to match the balanced and imbalanced classification. The congruent probability is the mean probability of congruent neurons (0° ~ 90°) from both balanced and imbalanced groups and the opposite probability from opposite neurons (90° ~ 180°) from both groups. To align with the scale of balanced and imbalanced encoding, we denote *ε* = 2*ε*′ when plotting the congruent-and-opposite inference efficiency in Figure 5(f).

### 4.9. Inference Based on a Fixed-Criterion Strategy

(22) $$\begin{array}{c} P\left(C=1\left|x_{\text{vis}},x_{\text{ves}}\right.\right)=\left\{\begin{array}{c} 1\text{ if }\left|x_{\text{vis}}-x_{\text{ves}}\right|+\xi <\kappa ,\\ 0\text{ if }\left|x_{\text{vis}}-x_{\text{ves}}\right|+\xi \geq \kappa , \end{array}\right.\;\xi \sim N\left(0,\sigma_{\text{noise}}\right). \end{array}$$

*x*_vis_ and *x*_ves_ are the visual and vestibular location samples from each Gaussian distribution, and |*x*_vis_ − *x*_ves_| corresponds to one sampling of ∆*θ*_uni_. The fixed-criterion (FC) strategy results in an inference based on the criterion *κ*, which is fixed and independent of the prior. The inference is binary: if the measured disparity |*x*_vis_ − *x*_ves_| is smaller than *κ*, the unit infers robust integration (*P*(*C* = 1|*x*_vis_, *x*_ves_) = 1); otherwise, it infers separation (*P*(*C* = 1|*x*_vis_, *x*_ves_) = 0). When noise is present, the disparity measurement is modified as |*x*_vis_ − *x*_ves_| + *ξ*, but *κ* remains fixed. In this case, both integration and separation inferences are possible if the noisy measurement approaches *κ*. The inference was repeated 100,000 times to represent the integration functions of MST-d neurons. When the integration functions were fitted, only *κ* was set as a free parameter.

### 4.10. Inference Based on a Bayesian Strategy

We assumed that the stimulus measurements would follow a Gaussian distribution with different mean positions (*x*_vis_ ~ *s*_vis_(*μ*_vis_, *σ*_vis_) and *x*_ves_ ~ *s*_ves_(*μ*_ves_, *σ*_ves_)), where *σ*_vis_ = *σ*_ves_ = *σ* is a free parameter. The Bayesian strategy computes the posterior probability based on the prior [46, 53, 54].

(23) $$\begin{array}{c} P\left(C=1\left|x_{\text{vis}},x_{\text{ves}}\right.\right)=\frac{P\left(x_{\text{vis}},x_{\text{ves}}\left|C=1\right.\right)P\left(C=1\right)}{P\left(x_{\text{vis}},x_{\text{ves}}\right)}. \end{array}$$

Aside from the sampling distribution *σ*, the common cause distribution and the prior probability *P*(*C* = 1) were also set as free parameters to provide an optimal fit for the biophysical simulation. To align the binary judgments, we adopted an optimal Bayesian reporter that reports integration if *P*(*C* = 1|*x*_vis_, *x*_ves_) > 0.5; otherwise, it reports separation [20, 21, 30]. As with the fixed-criterion strategy, reporting was repeated 100,000 times. Different integration functions and psychophysical functions were fitted by adjusting only the prior *P*(*C* = 1).

### 4.11. Psychophysical Decisions by Neural Monte Carlo Sampling

We adopted a biophysically plausible decision process that weighs the group response sampling of each type of multisensory encoder. The balanced group has been demonstrated to encode separation, and the imbalanced group has been demonstrated to encode integration. Considering both of them as computation bases, the decision neuron reported separation if the balanced group responded more strongly than the imbalanced group and vice versa. Since ∆*θ*_mul_ is characterized by distribution in both groups, the multisensory preference (∆*θ*_mul_) of the MST-d inputs may not be traversed, as the decision neuron does not necessarily receive many inputs under real conditions. Based on this fact, the decision neuron model performed probabilistic sampling according to the observed distribution of ∆*θ*_mul_ in the balanced (*D*_bal_) or imbalanced (*D*_imbal_) group; meanwhile, the input components remain proportional (balanced group: 70/115 = 0.61; imbalanced group: 45/115 = 0.39; balanced : imbalanced ≈ 3 : 2). For example, if the decision neuron received 15 inputs from the MST-d, then 9 of them were from balanced neurons and 6 were from imbalanced neurons. Among the 9 balanced neurons, the distribution of ∆*θ*_mul_ was fairly uniform for each neuron, and there was a fairly good chance that neurons with various disparities were represented. On the other hand, each of the 6 imbalanced neurons was more likely to sample a small ∆*θ*_mul_. The neuronal response was obtained from a data-averaged multisensory tuning function (*f*_bal_ and *f*_imbal_; see Supplementary Figure S2), which measures the difference between the preferred multisensory disparity and real input disparity (∆*θ*′). Finally, the decision neuron computed the summed responses and decided which response was higher. We repeated such decision reports for 100,000 trials in each real input disparity condition.

(24) $$\begin{array}{c} ∆\theta \prime_{i}=∆\theta_{\text{input}}-∆\theta_{i},\text{where}∆\theta_{i}\in D_{\text{bal}},\\ ∆\theta \prime_{j}=∆\theta_{\text{input}}-∆\theta_{j},\text{where}∆\theta_{j}\in D_{\text{imbal}},\\ R_{\text{bal}}^{i}=f_{\text{bal}}\left(∆\theta \prime_{i}\right).\\ R_{\text{imbal}}^{j}=f_{\text{imbal}}\left(∆\theta \prime_{j}\right).\\ \text{Decision}=\overset{n_{\text{bal}}}{\underset{i=1}{\sum }}R_{\text{bal}}^{i}-\overset{n_{\text{imbal}}}{\underset{j=1}{\sum }}R_{\text{imbal}}^{j}\left\{\begin{array}{c} \geq 0⟶S\text{eparation}\\ <0⟶\text{Integration} \end{array}\right.. \end{array}$$

## Figures and Tables

**Table 1:** Model parameters.

Lateral connections (Mexican hat)				External inputs		Forward connections		Intrinsic noise	
*w*_ex_	*σ*_ex_	*w*_in_	*σ*_in_	*w*_external_	*σ*_external_	*w* ^MT^ and *w*^PIVC^	*σ*_forward_	*μ*	*σ*_noise_
5	10	3	70	25	160	Follow data distribution of ratio (*r*)	5	0	6

## Data Availability

The datasets generated during and/or analysed during the current study are available from the corresponding author on reasonable request.

## Additional Points

*Code Availability.* The key analysis codes are available for reasonable request.

## Supplementary Materials

Supplementary Materials Figure S1: model simulation of response curves of MST-d neuron. Figure S2: data-derived multisensory tuning functions of balanced and imbalanced groups. Figure S3: stochastic resonance elicited by noise following a uniform distribution. Figure S4: simulated decision with varying category proportions. Figure S5: distribution of congruent and opposite neurons in balanced and imbalanced categories. Figure S6: simulation from uniform to polarized (skewed) computational bases in a decision role.
