# Supplementary material for: Unveiling Dynamic System Strategies for Multisensory Processing: From Neuronal Fixed-Criterion Integration to Population Bayesian Inference
Source: Research (Wash D C). 2022 Aug 19;2022:9787040. doi: 10.34133/2022/9787040 (PMC9422331; doi:10.34133/2022/9787040)
Supplement: Supplementary Materials — Figure S1: model simulation of response curves of MST-d neuron. Figure S2: data-derived multisensory tuning functions of balanced and imbalanced groups. Figure S3: stochastic resonance elicited by noise following a uniform distribution. Figure S4: simulated decision with varying category proportions. Figure S5: distribution of congruent and opposite neurons in balanced and imbalanced categories. Figure S6: simulation from uniform to polarized (skewed) computational bases in a decision role. [file 9787040.f1.zip › Jiawei_RESEARCH-supplementary-20220530.docx]

**A. Model simulation of response curves of MST-d neuron**


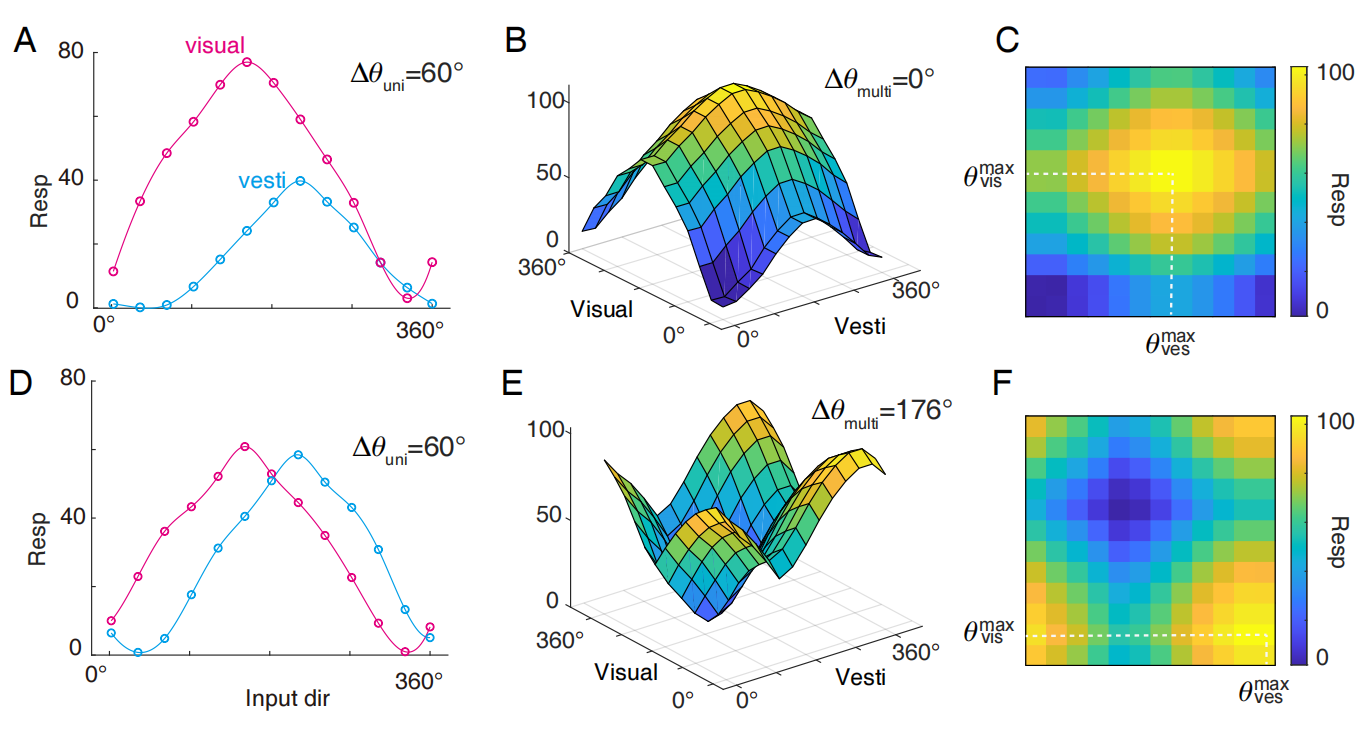


**Fig. S1 Model simulation of response curves of MST-d neuron. A** Unisensory response curve of an imbalanced neuron. **B** Multisensory response grid of the same neuron. **C** Pseudo-colour plot of the same grid in B. **D, E, F** same as A, B, and C of a balanced neuron.

**B. multisensory tuning functions**


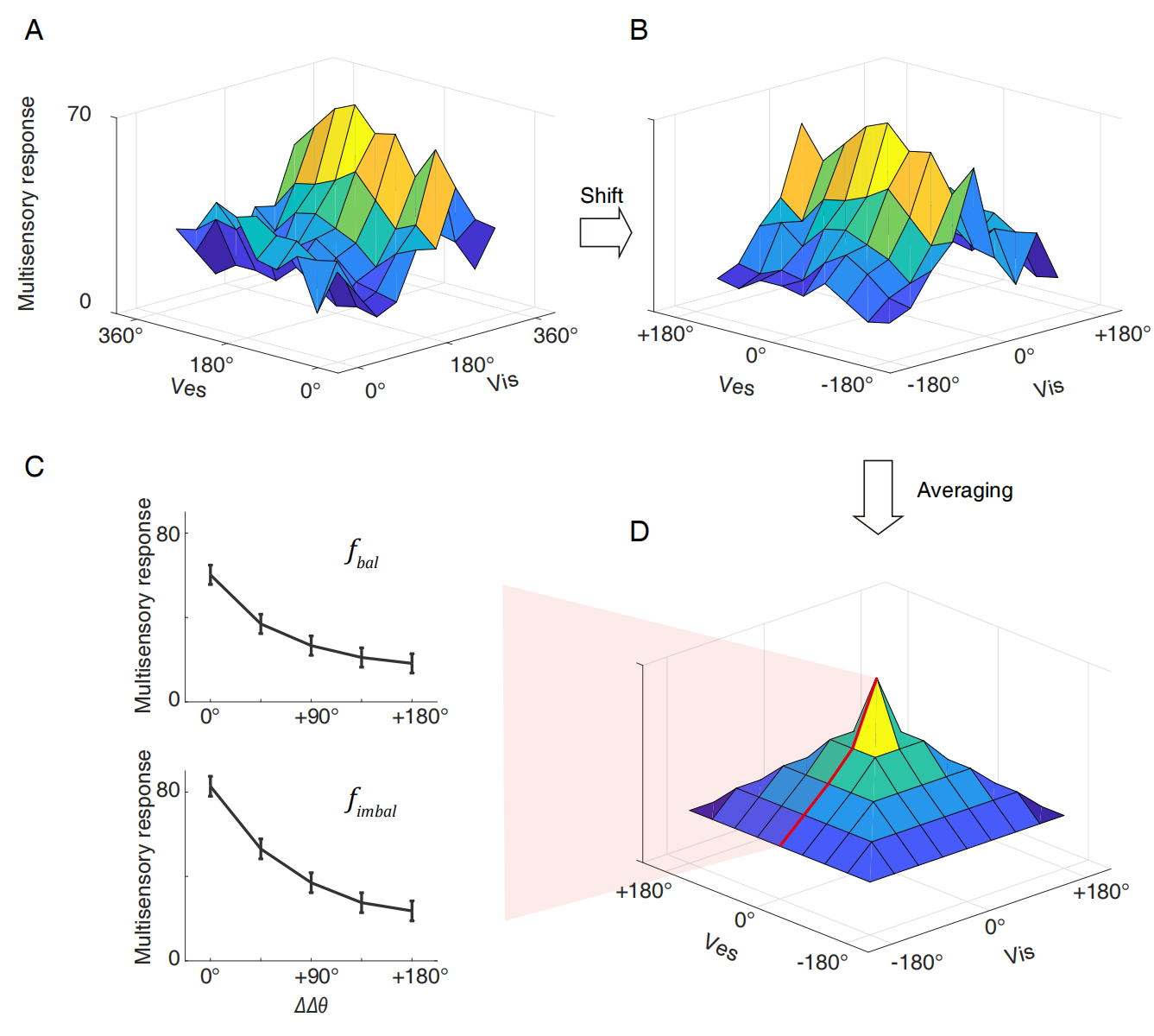


**Fig. S2 Data-derived multisensory tuning functions of balanced and imbalanced groups. A** The original multisensory tuning grid as a function of visual and vestibular directions. **B** Shifted tuning grid from A, where the multisensory-preferred response lies at [0$^{\circ}, 0^{\circ}$] in the grid. **C** Multisensory tuning response by disparity difference ($\Delta\theta'={|\Delta\theta}_{cue}-{\Delta\theta}_{mul}|$). The bar shows the mean standard deviation, which is averaged from all multisensory responses of all neurons in the group ($8\times8\times70$ in the balanced group and $8\times8\times45$ in the imbalanced group). In the balanced group, the standard deviation of multisensory responses was 4.58 spikes/s, and in the imbalanced group, the value was 4.72 spikes/s. **D** Normalized tuning grid from B. The peripheral responses that are equidistant from the center are averaged as one response. Therefore, the two-dimensional multisensory responses are transferred into one dimension, as shown in C.

**C. Noise as random number sequence in the model**

When the noise in the CANN model is replaced by a random number sequence with uniform distribution, the noise intensity became more stable, thus the stochastic resonance is elicited by larger noise intensity ($\sigma$=18). Accordingly, the encoding of congruent and opposite neurons achieves maximal efficiency when noise intensity $\sigma$=1. When fitting the CANN model with noise as random number sequence, the model provides equally-good fit of data as Gaussian noise. The best-fit noise level is $\sigma_{best-fit}=20$, which is still close to the optimal level to elicit stochastic resonance.


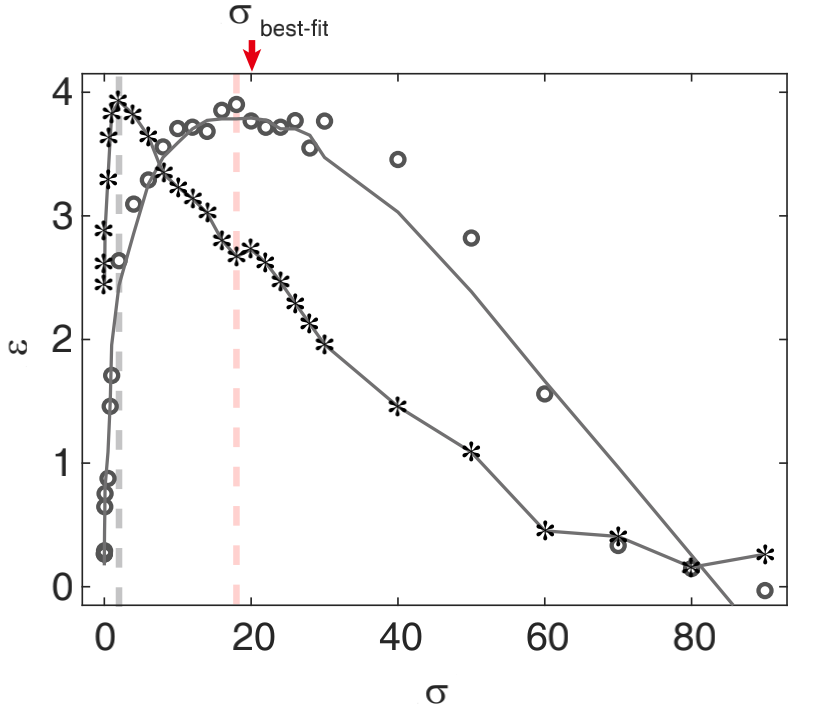


**Fig. S3 Stochastic resonance elicited by noise following a uniform distribution.** The noise is described as $\sigma\cdot\gamma$, where $\gamma$ is a random number sequence ranged in [-1, 1] with uniform distribution. $\sigma$ denotes the noise intensity. $\varepsilon$ denotes inference efficiency, which for balanced and imbalanced neurons is computed by Eq.(25) (circles), and for congruent and opposite neurons is computed by Eq.(26) (asterisks).

**D. Simulated decision with varying category proportions**

In the main results, we presented that neuronal responses between balanced and imbalanced categories can produce Bayesian decisions. In this section, we present another principle that can produce the decision flexibility, the principle is varying category proportions. In physiological conditions, the number of neurons that send output to downstream could vary even when the synaptic plasticity is not considered. This is due to that real neurons have various response thresholds, thus different input intensity alters the number of responding neurons. To balanced and imbalanced neurons, if the dominant input is strong, then it is likely that imbalanced neurons with correspondingly-dominant preference counts more than the default proportion ($n_{bal}:n_{imbal}<3:2$). On the other hand, if the two inputs are strong and equally strong, then the number of activated balanced neurons can outweigh that of imbalanced neurons ($n_{bal}:n_{imbal}>3:2$). We simulated a minimal change (one neuron in total sample size as 15, which is constant), and we presented that the change of neuronal category proportion produced drastic decision bias to either reporting common source (integration) or different sources (separation).


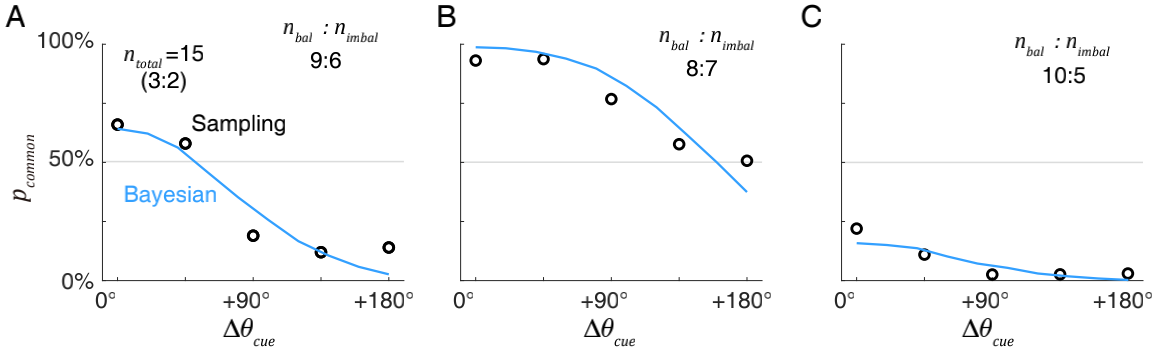


**Fig. S4 simulated decision with varying category proportions. A** Decision of whether to report a common source or different sources, shown as probability ($p_{common}$). Decisions were simulated by Monte-Carlo sampling (circles) from balanced and imbalanced categories and repeated 10,000 times. Solid curves denoted Bayesian strategy. **B** decision probability when changing the proportion between the two categories from 9:6 to 8:7, which case simulated that imbalanced neurons are more activated. **C** decision probability when changing the proportion to 10:5, which case simulated that balanced neurons are more activated. The best-fit prior in three conditions are 0.46, 0.87 and 0.39, while other parameters held constant.

**E. congruent and opposite neuronal category**

Previous work proved that multisensory computation of integration and separation can be approximated to vector computation, where congruent preferences encode integration and opposite preferences encode separation. Here, we demonstrated the distribution of congruent and opposite neuron occurrence in balanced and imbalanced neuronal category as below.


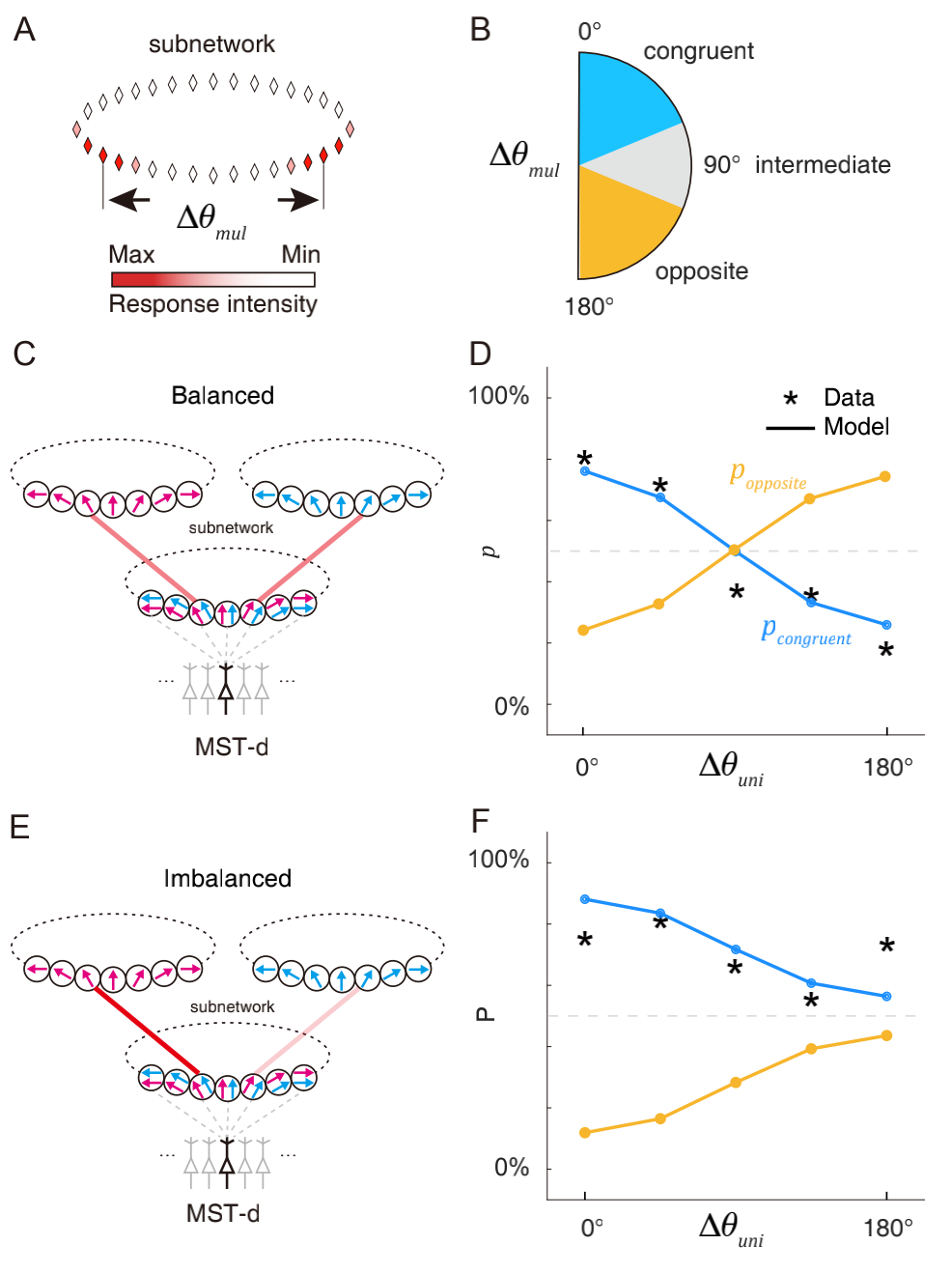


**Fig. S5 Distribution of congruent and opposite neurons in balanced and imbalanced categories. A** Schematic of ${\Delta\theta}_{mul}$, characterized by the distance between responding groups on the subnetwork. **B** The definition of congruent and opposite neurons. Due to the discrete 45-degree experimental operations, each measurement contains ${\Delta\theta}_{mul}\pm22.5^{\circ}$. Therefore, the congruent neurons are those with $0\leq{\Delta\theta}_{mul}\leq67.5^{\circ}$, opposite neurons are those with $112.5^{\circ}\leq{\Delta\theta}_{mul}\leq180^{\circ}$, and those in between are intermediate neurons. **C** Schematic of balanced neuron simulations. **D** Probabilistic distribution of congruent and opposite neurons. $p_{congruent}={n_{congruent}}/{(n_{congruent}+n_{opposite})}$. **E,F** same as C,D but for imbalanced neurons.

**F. Specific-distributed bases produces maximal decision flexibility**

It is intuitively surprising that a flexible decision is made from the comparison of the balanced and imbalanced group responses as computational bases, between which the ${}_{mul}$ distributions are not clearly polarized but have a wide range of overlap. Such widely distributed bases are usually neglected because most works have focused on interpreting decisions by polarized bases such as congruent and opposite neurons (**SFig.6A**). To compare the bases, we also simulated the decision in the condition where the balanced group contained only neurons with high disparity, while the imbalanced group contained only neurons with low disparity, and we performed the same psychophysical decisions as mentioned before. The total inputs are 100 MST-d samplings, and the proportion changes parallels Supplementary Fig.5, except that we increased the total sample size to 100. The results showed that even though the prior is introduced by proportion change as well, such polarized bases do not make decisions as flexibly as the balanced and imbalanced groups; instead, they perform rigid decisions that resemble FC strategy (**SFig.6B**). Only when the proportion itself changed significantly were the decisions altered therewith (result not shown). This means that the relationship to the probabilistic distribution of ${}_{mul}$ is critical to flexible Bayesian computation in neural systems.

We further simulated different pairings of distribution skewness (**SFig.6C**). According to the analysis above, the balanced neurons mostly performed separation and had larger ${}_{mul}$, while the imbalanced neurons mostly performed integration and had small ${}_{mul}$. Therefore, the balanced group was set to gradually skew towards 180$^{\circ}$, and the imbalanced group gradually skewed towards 0$^{\circ}$. The more skewed the distributions were, the more polarized the bases. Decision flexibility was measured by inference efficiency ($\varepsilon$) as above (see Methods). **SFig.6D** indicates that the maximum efficiency was produced when the balanced distribution was uniform. At the same time, the imbalanced distribution was moderately skewed toward small values of ${}_{mul}$ just as seen in the empirical data. In other words, a flexible decision required both uniformly-distributed separation encoders and skewed integration encoders. If the integration encoders were extremely skewed, the flexibility deteriorates because they responded in the same pattern, and magnifying their weights did not increase the flexibility of the decision process. Furthermore, if both encoders were polarized as in **SFig.6A**, the decision strategy approximated the FC strategy, which is inflexible and rigid. These results also explained the ${}_{uni}$ distribution in the physiological recordings. At first it seems contradictory that the balanced group tended to perform separation despite its ${}_{uni}$ being dominated by congruent neurons, while the imbalanced group tended to perform integration despite its ${}_{uni}$ being dominated by opposite neurons. When the results are combined, it is obvious that the ${}_{uni}$ distribution serves to produce the specific ${}_{mul}$ distribution, which makes the decision maximally flexible.


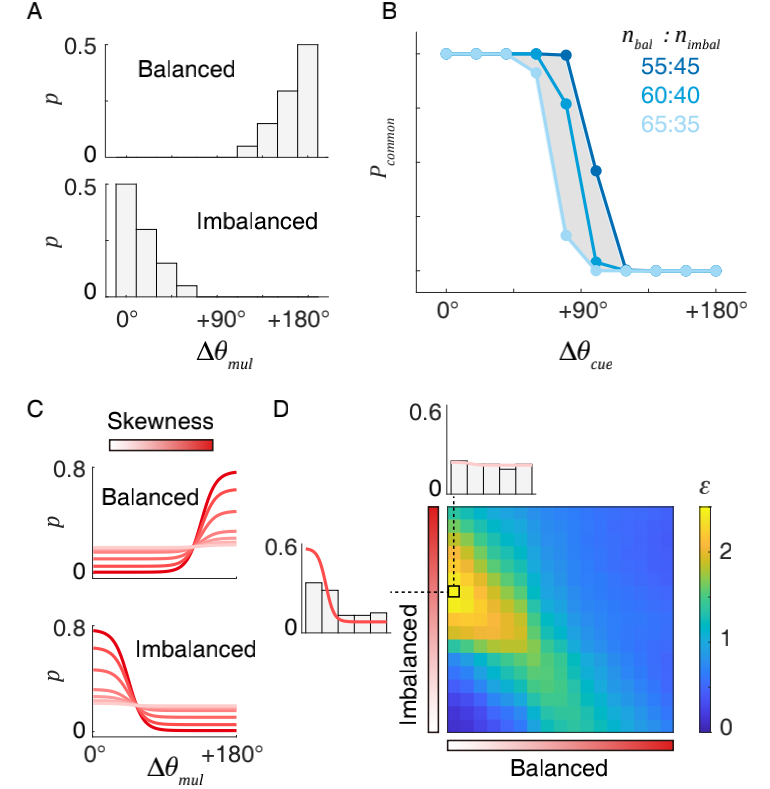


**Fig. S6 Simulation from uniform to polarized (skewed) computational bases in a decision role. A** Examples of the ${\Delta\theta}_{mul}$ distributions of balanced and imbalanced groups, which are skewed in opposite directions in accordance with the polarized nature of the neuronal bases. **B** The biophysical decision function derived from 100,000 decision neurons, each of which receives 100 inputs from the MST-d with the distributions shown in A. The grey shading indicates the flexible inference range, which can be measured by inference efficiency. **C** The skewness gradient of ${\Delta\theta}_{mul}$ distributions in the balanced and imbalanced bases. The more skewed the distribution, the more polarized the bases. **D** Inference efficiency as a function of the skewness of both bases. The skewness that produced the maximum inference efficiency was compared with the physiological data distribution, as shown on the left and top.
