# Supplementary figures and images for: Unveiling Dynamic System Strategies for Multisensory Processing: From Neuronal Fixed-Criterion Integration to Population Bayesian Inference
Source: Research (Wash D C). 2022 Aug 19;2022:9787040. doi: 10.34133/2022/9787040 (PMC9422331; doi:10.34133/2022/9787040)

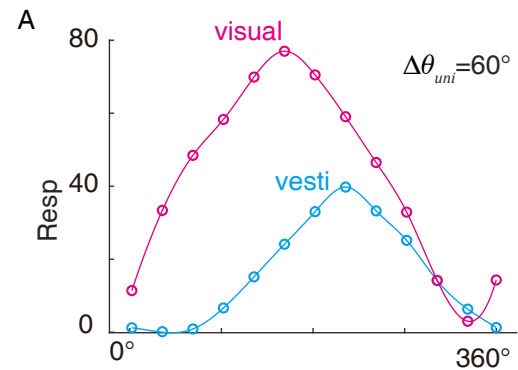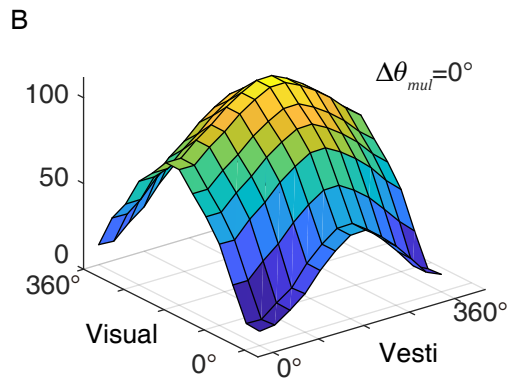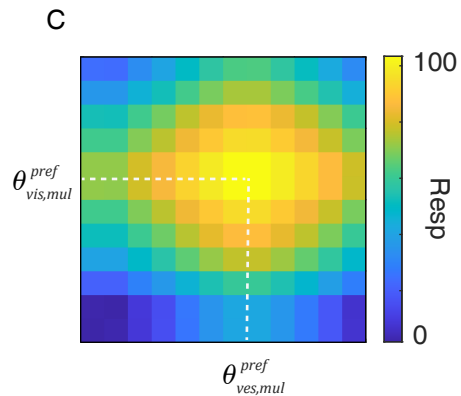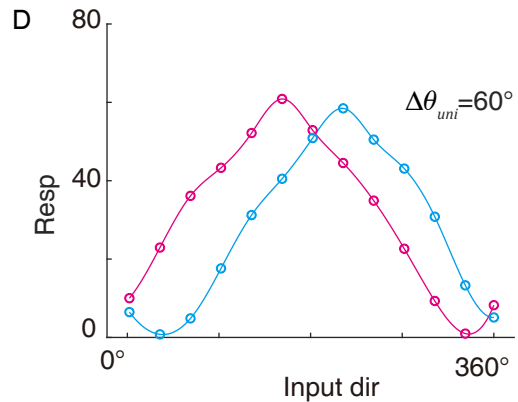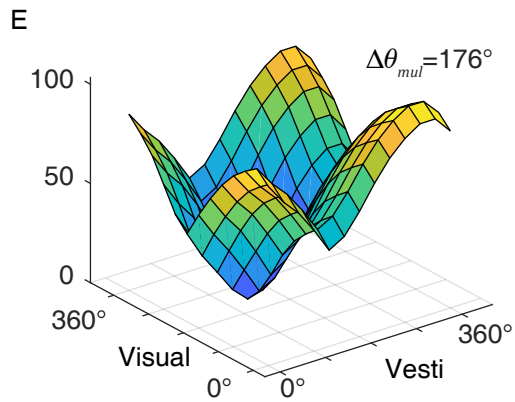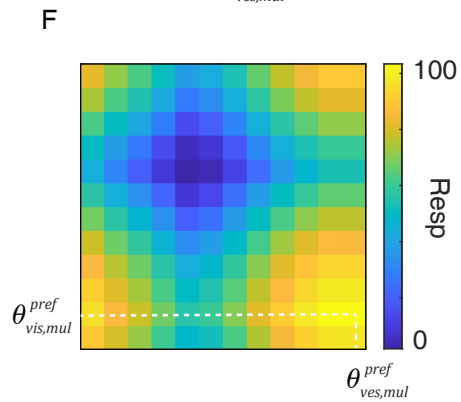

Supplement: Supplementary Materials — Figure S1: model simulation of response curves of MST-d neuron. Figure S2: data-derived multisensory tuning functions of balanced and imbalanced groups. Figure S3: stochastic resonance elicited by noise following a uniform distribution. Figure S4: simulated decision with varying category proportions. Figure S5: distribution of congruent and opposite neurons in balanced and imbalanced categories. Figure S6: simulation from uniform to polarized (skewed) computational bases in a decision role. [file 9787040.f1.zip › supplementary fig.1-research-20220710.pdf]

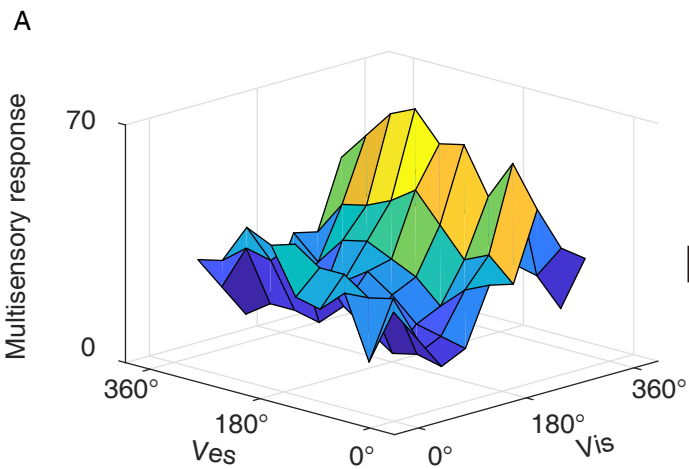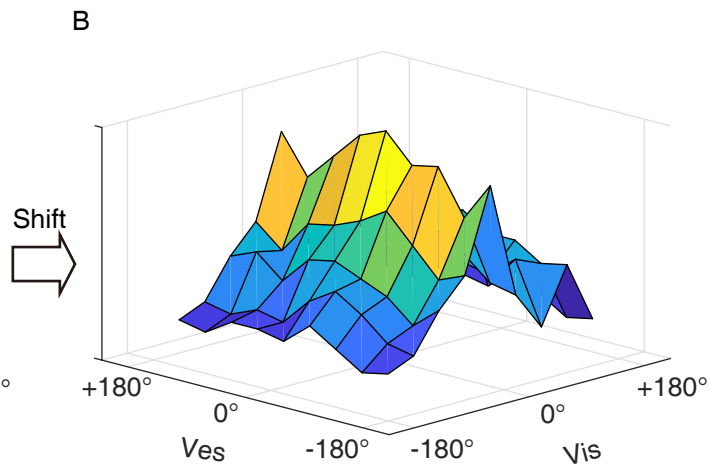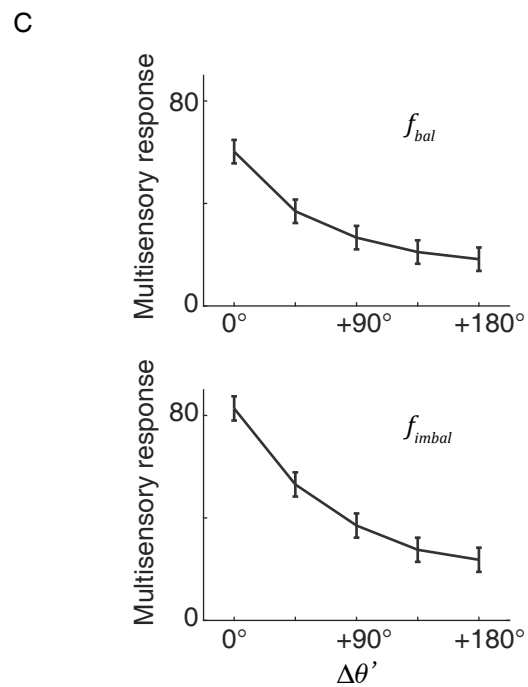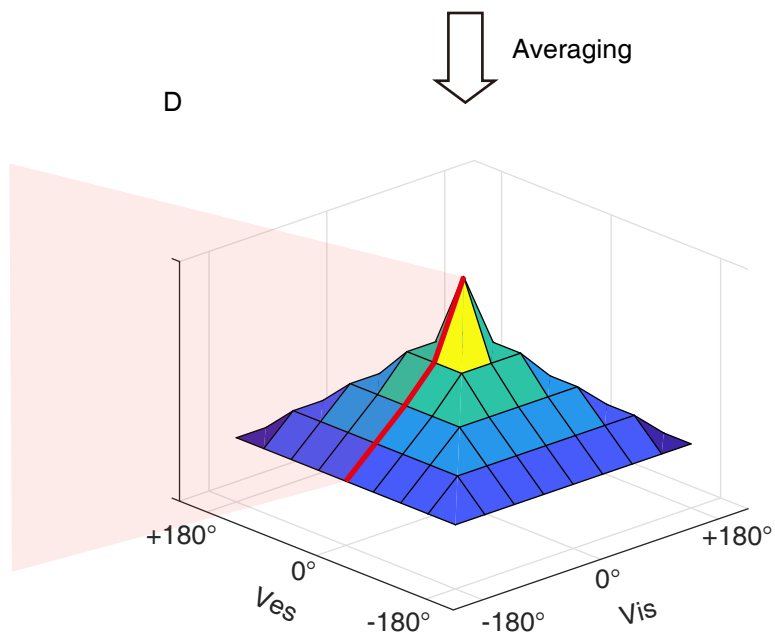

Supplement: Supplementary Materials — Figure S1: model simulation of response curves of MST-d neuron. Figure S2: data-derived multisensory tuning functions of balanced and imbalanced groups. Figure S3: stochastic resonance elicited by noise following a uniform distribution. Figure S4: simulated decision with varying category proportions. Figure S5: distribution of congruent and opposite neurons in balanced and imbalanced categories. Figure S6: simulation from uniform to polarized (skewed) computational bases in a decision role. [file 9787040.f1.zip › supplementary fig.2-research-20220710.pdf]

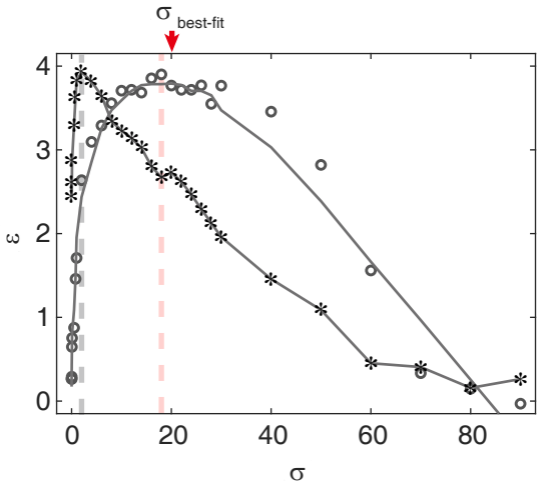

Supplement: Supplementary Materials — Figure S1: model simulation of response curves of MST-d neuron. Figure S2: data-derived multisensory tuning functions of balanced and imbalanced groups. Figure S3: stochastic resonance elicited by noise following a uniform distribution. Figure S4: simulated decision with varying category proportions. Figure S5: distribution of congruent and opposite neurons in balanced and imbalanced categories. Figure S6: simulation from uniform to polarized (skewed) computational bases in a decision role. [file 9787040.f1.zip › supplementary fig.3-research-20220710.pdf]

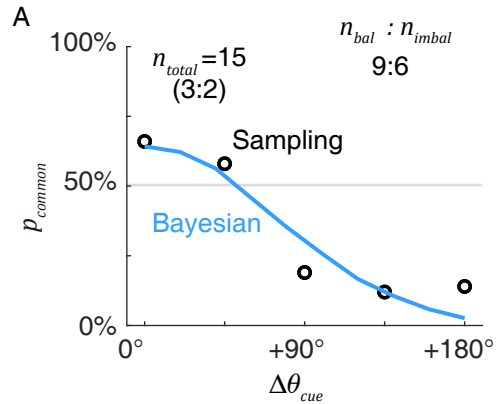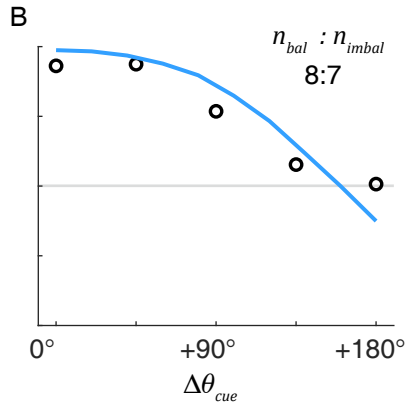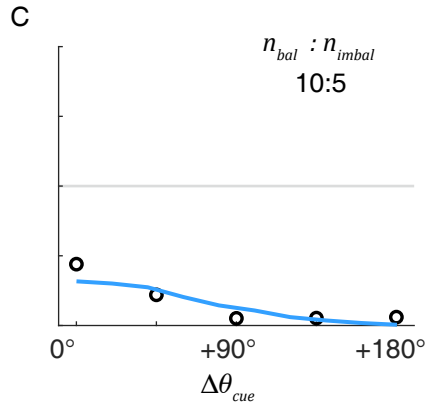

Supplement: Supplementary Materials — Figure S1: model simulation of response curves of MST-d neuron. Figure S2: data-derived multisensory tuning functions of balanced and imbalanced groups. Figure S3: stochastic resonance elicited by noise following a uniform distribution. Figure S4: simulated decision with varying category proportions. Figure S5: distribution of congruent and opposite neurons in balanced and imbalanced categories. Figure S6: simulation from uniform to polarized (skewed) computational bases in a decision role. [file 9787040.f1.zip › supplementary fig.4-research-20220710.pdf]

A

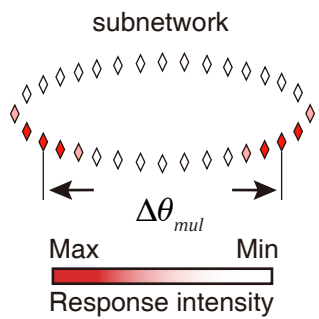

B

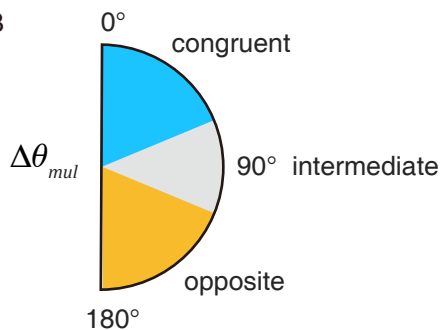

C

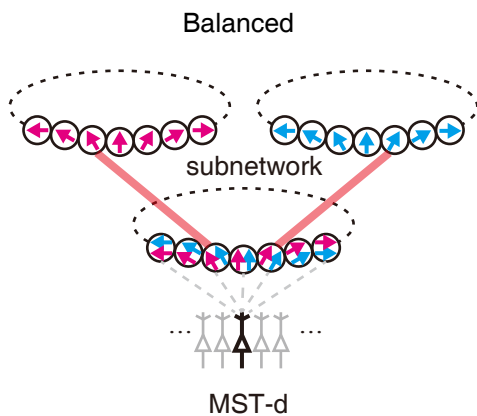

D

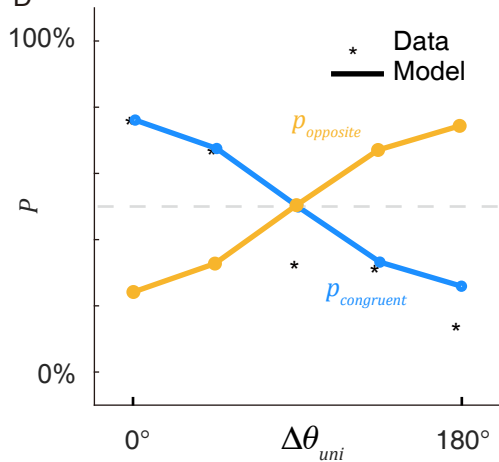

E

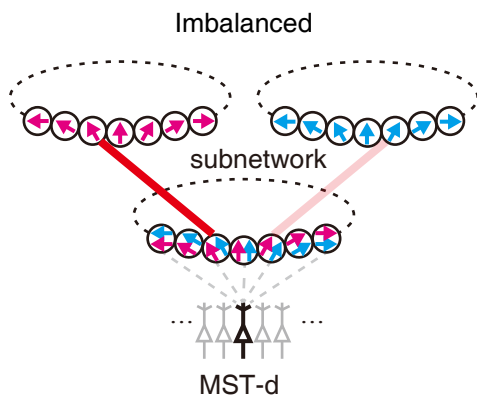

F

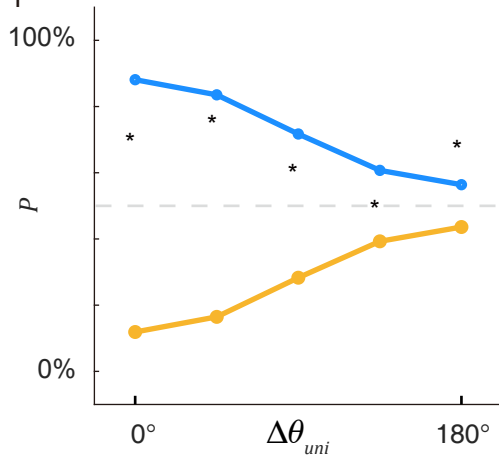

Supplement: Supplementary Materials — Figure S1: model simulation of response curves of MST-d neuron. Figure S2: data-derived multisensory tuning functions of balanced and imbalanced groups. Figure S3: stochastic resonance elicited by noise following a uniform distribution. Figure S4: simulated decision with varying category proportions. Figure S5: distribution of congruent and opposite neurons in balanced and imbalanced categories. Figure S6: simulation from uniform to polarized (skewed) computational bases in a decision role. [file 9787040.f1.zip › supplementary fig.5-research-20220710.pdf]

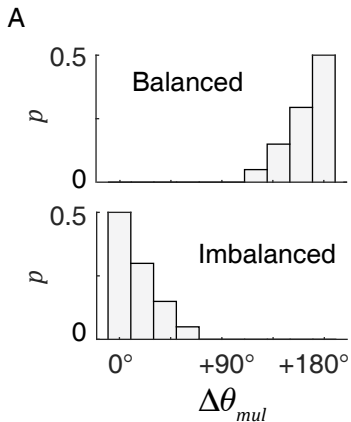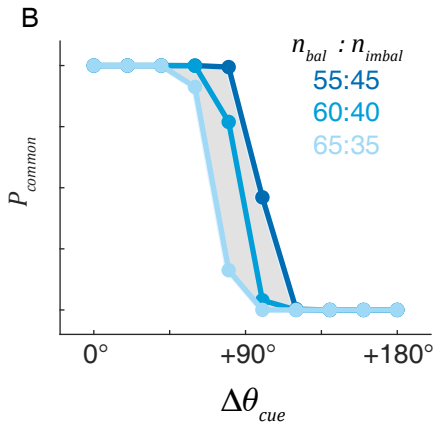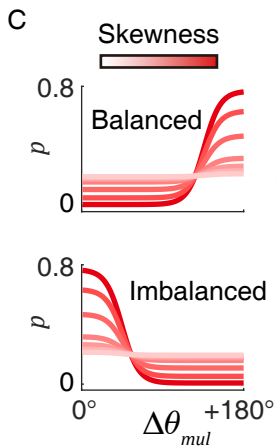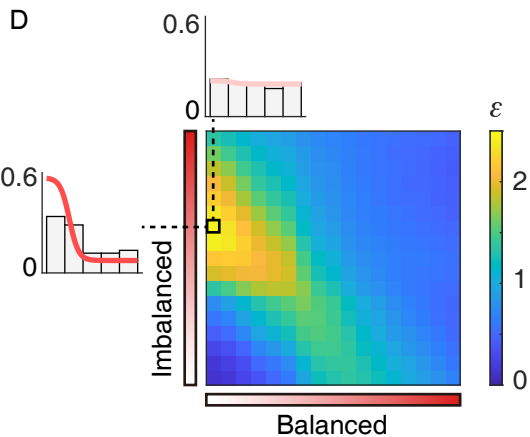

Supplement: Supplementary Materials — Figure S1: model simulation of response curves of MST-d neuron. Figure S2: data-derived multisensory tuning functions of balanced and imbalanced groups. Figure S3: stochastic resonance elicited by noise following a uniform distribution. Figure S4: simulated decision with varying category proportions. Figure S5: distribution of congruent and opposite neurons in balanced and imbalanced categories. Figure S6: simulation from uniform to polarized (skewed) computational bases in a decision role. [file 9787040.f1.zip › supplementary fig.6-research-20220710.pdf]
